# Lanthanide Complexes (Gd^III^ and Eu^III^) Based on a DOTA‐TEMPO Platform for Redox Monitoring via Relaxivity

**DOI:** 10.1002/asia.202200544

**Published:** 2022-07-28

**Authors:** Richard Barré, Damien Mouchel dit Leguerrier, Quentin Ruet, Lionel Fedele, Daniel Imbert, Véronique Martel‐Frachet, Pascal H. Fries, Jennifer K. Molloy, Fabrice Thomas

**Affiliations:** ^1^ Univ. Grenoble Alpes CNRS DCM 38000 Grenoble France; ^2^ Institute for Advanced Biosciences INSERM U1209 UMR CNRS 5309 Grenoble Alpes University 38700 La Tronche France; ^3^ EPHE PSL Research University 75014 Paris France; ^4^ Univ. Grenoble Alpes CEA CNRS IRIG-LCBM 38000 Grenoble France; ^5^ Univ. Grenoble Alpes CEA CNRS IRIG-SYMMES 38000 Grenoble France

**Keywords:** gadolinium, nitroxide, contrast agent, redox, relaxivity

## Abstract

Three lanthanide complexes (Ln=Gd, Eu) based on a DO3 A (**[Ln(L_1_)]**) or DO2 A (**[Ln(L_2–3_)]^+^**) platform appended by a redox active TEMPO‐based arm were prepared. Complex **[Ln(L_2_)]^+^** shows an alkyne arm, offering the possibility of postfunctionalization by click reaction to yield **[Ln(L_3_)]^+^**. The complexes demonstrate a redox response whereby the hydroxylamine, nitroxide and oxoammonium forms of the arm can be obtained in turn. Luminescence measurements on the europium complexes support an octadentate (**L_1_**, **L_3_**) or heptadentate (**L_2_**) chelation by the ligand, with one water molecule in the inner coordination sphere. The relaxivity was determined from 20 kHz to 30 MHz by fast‐field cycling NMR. The three Gd^III^ complexes under their hydroxylamine form **[Gd(L_1_)]** and **[Gd(L_2–3_)]^+^** show *r_1_* values of 7.0, 5.1 and 5.0 mM^−1^ s^−1^ (30 KHz), which increase to 8.8, 5.5 and 6.1 mM^−1^ s^−1^ in the nitroxide form. The radical complexes are not toxic against M21 cell lines, at least up to 40 μM. By using EPR spectroscopy we establish that they do not penetrate the cells with the exception of **[Eu(L_2_)]^+^**.

## Graphical abstract

**The reported gadolinium complexes** feature one coordinated water molecule, and the relaxivity increases upon oxidation of the hydroxylamine form of the TEMPO unit into the corresponding nitroxide radical.

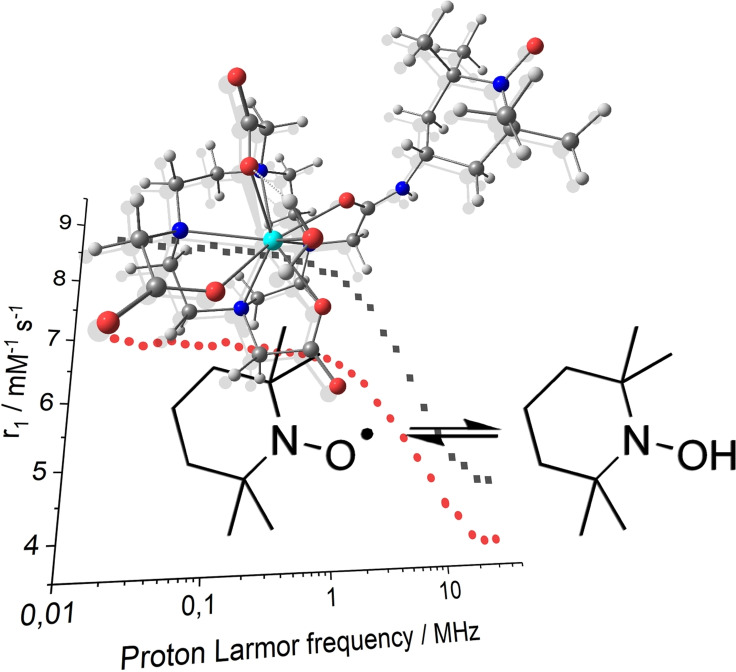

## Introduction

The disruption of the natural metabolism of oxygen has an important role in cell signalling and cellular homeostasis.^[1]^ This disruption induces a redox misbalance that may cause the over expression of reactive oxygen species (ROS).[^1b^, ^2^] Most of the ROS are toxic and capable of damaging almost all the biomolecules. Furthermore these species have been shown to be important in the proliferation of many diseases such as cancers,^[3]^ Alzheimer's^[4]^ etc. Hence, the direct and non‐invasive redox imaging of tissues can constitute an innovative tool for diagnostics.^[5]^

Lanthanide ions possess ideal properties to be exploited in imaging and detection due to their peculiar photophysical and magnetic signatures.^[6]^ Indeed, their narrow emission lines, combined with both long lifetimes of their excited states and the possibility to modulate the emission wavelength by permutation of the metal confers them decisive advantages over classical fluorophores.^[7]^ Moreover, the high number of unpaired electrons on Gd^3+^, makes it ideal for designing contrast agents (CAs). Dotarem, Gadovist, ProHance, which are all gadolinated MRI (magnetic resonance imaging) CAs, are for instance widely used clinically.^[8]^ However, the stable +III redox state of most of the lanthanide cations precludes their use as redox probes.^[5]^ Two strategies have emerged in the literature to overcome the redox‐inertness of the lanthanide ions.^[9]^ One consists in designing a ligand prone to chemical transformations, usually irreversible, promoted by specific ROS. As an example, the anthryl moiety reacts with ^1^O_2_ to give endoperoxides,^[10]^ altering the sensitization process or the CEST signal^[11]^ in Eu^3+^ complexes. On the other hand, benzyl boronic acids are typically cleaved^[12]^ or undergo transformations^[8]^ in the presence of H_2_O_2_ with further change in sensitization. Similarly, trimesamide derivatives get oxidized by OH⋅ into efficient antennas for the Tb^3+^ ion, affording a switch‐on probe for this ROS.^[13]^ In a different approach, yet aimed at detecting a change in redox status, we designed an iminohydroxylamine‐appended DOTA ligand.^[14]^ Its lanthanide complexes demonstrated trapping of hydroxyl radicals, followed via electron paramagnetic resonance (EPR).^[14]^ An alternative strategy consists in using a redox active ligand capable of responding to changes in potential or to the presence of ROS by switching between oxidised and reduced states (1 or 2 electrons exchange). When both redox states exhibit different magnetic or optical signature, molecular weight or coordination sphere one may expect changes in the lanthanide properties. For example, we demonstrated a change of more than 90% in the luminescent properties of Yb^III^ using switchable redox active phenolate‐containing ligands,^[15]^ while Faulkner et al. observed a quenching of the luminescence by about 25% upon oxidation of ferrocene functionalized Yb^III^ chelates.^[16]^

The propensity of nitroxide moieties to reflect redox changes in biological conditions by chemical exchange saturation (CEST) were documented recently in europium complexes.^[17]^ It is basically based on the bistability of this unit, which can exist either under its nitroxide radical or its one‐electron reduced hydroxylamine form in biological fluids.^[18]^

**Figure 1:**
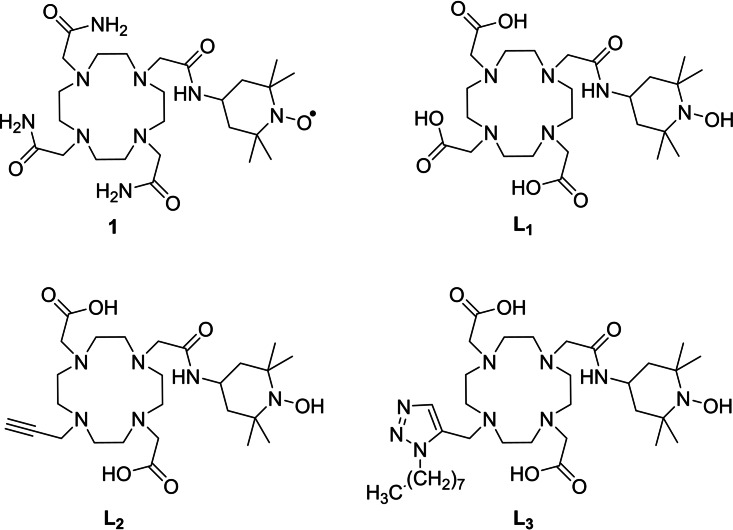
Structure of ligands of interest. **L_1_–L_3_** are depicted under their hydroxylamine form.

Following our recent report on CEST‐active Eu^3+^ chelates appended by a nitroxide moiety (**1**, Figure 1)^[17a]^ we herein report a series of Ln complexes (Ln=Gd^3+^, Eu^3+^) based on a cyclen scaffold containing the redox active reduced TEMPO arm (Figure 1). Three derivatives were prepared, **L_1_** from DO3A and containing a single nitroxide, **L_2_** adding an alkyne (from DO2A) and **L_3_** from **L_2_** by a click reaction with an alkane chain as a proof of concept for the future functionalization with biomolecules. Electrochemical, EPR, luminescence and relaxivity studies have demonstrated specific redox sensitive activity.

## Results and Discussion

### General procedure for synthesis of the ligands and complexes

**Scheme 1:**
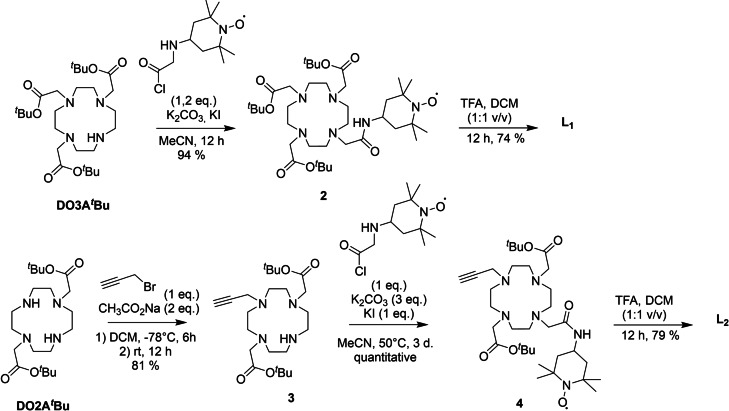
Synthetic pathway towards the ligands **L_1_** and **L_2_**.

The ligand **L_1_** was synthesised via nucleophilic substitution of commercial 1,4,7‐tris(*tert*‐butoxycarbonylmethyl)‐1,4,7,10‐tetraazacyclododecane (DO3A^*t*^Bu) with 4‐(2‐chloroacetamido)‐2‐2’‐6‐6’‐tetramethylpyperidine‐1‐oxyl^[17a]^ to give intermediate **2**. Further deprotection using an equimolar mixture of trifluoroacetic acid and dichloromethane (Scheme 1) affords **L_1_** in an overall yield of 44%. Note that **L_1_** proved to be diamagnetic, with sharp resonances in its NMR spectrum (Figure S1) and no EPR signal. In particular, two resonances are observed at 1.46 and 1.52 ppm in the ^1^H NMR spectrum, which account for six protons each and correspond to the four methyl groups of a diamagnetic form of the TEMPO arm. This result is in line with the known sensitivity of TEMPO radicals to acidic conditions, whereby the nitroxide undergoes disproportionation into diamagnetic oxo‐ammonium and protonated hydroxylamine^[19]^ and possible thermal reduction of the first into hydroxylamines.^[20]^ Note that the nitroxide form can be recovered by comproportionation (see below).^[20]^

Ligand **L_2_** was synthesized in three steps from DO2A^*t*^Bu. The first step is a nucleophilic substitution of one amine from the macrocycle by propargyl bromide, which affords intermediate **3** (scheme 1). It is followed by a nucleophilic substitution of the 4‐(2‐chloroacetamido)‐2‐2’‐6‐6’‐tetramethylpyperidine‐1‐oxyl, giving intermediate **4**) and deprotection using the same procedure as for **L_1_** (Scheme 1). As for **L_1_** the ligand **L_2_** was isolated under a diamagnetic form (Figure S2).

Complexation was performed by mixing an equimolar amount of the ligand **L_1_** or **L_2_** and the lanthanide salts (Gd(OTf)_3_ or EuCl_3_) in H_2_O at neutral pH. After two days stirring at room temperature the complexes were precipitated in MeOH/Et_2_O, to give **[Ln(L_1_)]** and **[Ln(L_2_)]^+^** as their respective salts (Ln=Gd, Eu). The HR‐MS confirms their isolation by peaks at m/z=735.2194 (**[Gd(L_1_)]**+H]^+^), 706.2331 (**[Eu(L_1_)]**+H]^+^) (Figure S5), 693.24857 (**[Gd(L_2_)]^+^** +H]^+^) and 689.25312 (**[Eu(L_2_)]^+^** +H]^+^) (Figure S6) for the two chelates.

**Scheme 2:**
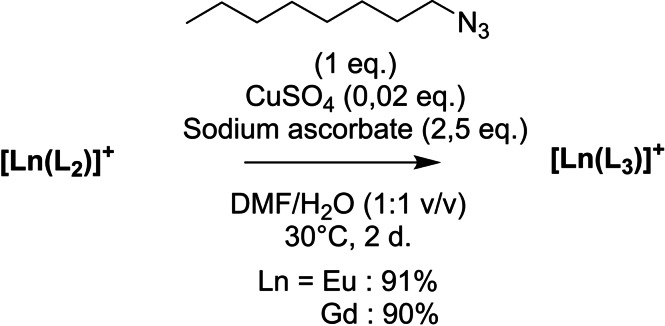
Synthetic pathway towards complexes [Ln(L_3_)]^+^.

The alkyne unit of **L_2_** has a dual function, to increase the charge on the complex and allow a further functionalization via a click cycloaddition. We investigated the feasibility of the reaction by introducing an octane chain on the complexes to yield **[Gd(L_3_)]^+^** and **[Eu(L_3_)]^+^**, with the aim of modulating its cell penetrating ability through a change in lipophilicity (Scheme 2). The reaction was performed in a DMF:H_2_O (50 : 50) mixture, in the presence of catalytic amount of Cu(II) sulphate, an excess (2.5 molar equivalents with respect to the complex) of ascorbate and 2 molar equivalents of 1‐azidooctane. The excess of ascorbate was used to both reduce Cu(II) sulphate and prevent Cu(II) promoted oxidation of the hydroxylamine. After two days the reaction did not evolve further and the complexes were isolated by precipitation. The **[Ln(L_3_)]^+^** complexes display the expected features in HR‐MS at 425.20282 (m/z=[**[Gd(L_3_)]^+^** +H]^2+^) and 422.70138 (m/z=[**[Eu(L_3_)]^+^** +H]^2+^) (Figure S7).

### Electrochemistry

The electrochemical behaviour of the ligands and complexes has been investigated by cyclic voltammetry (CV) in 0.5 mM acetonitrile solution using tetra‐*n*‐butyl‐ammonium perchlorate (TBAP) 0.1 M as electrolyte.

**Scheme 3:**
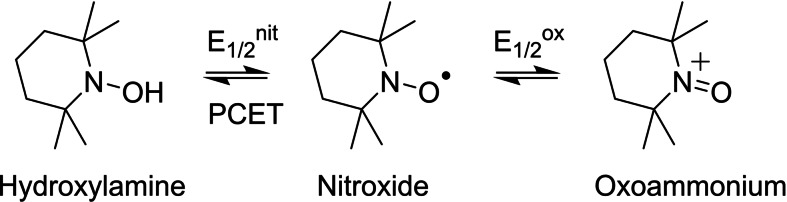
Redox chemistry of the TEMPO unit.

**Figure 2:**
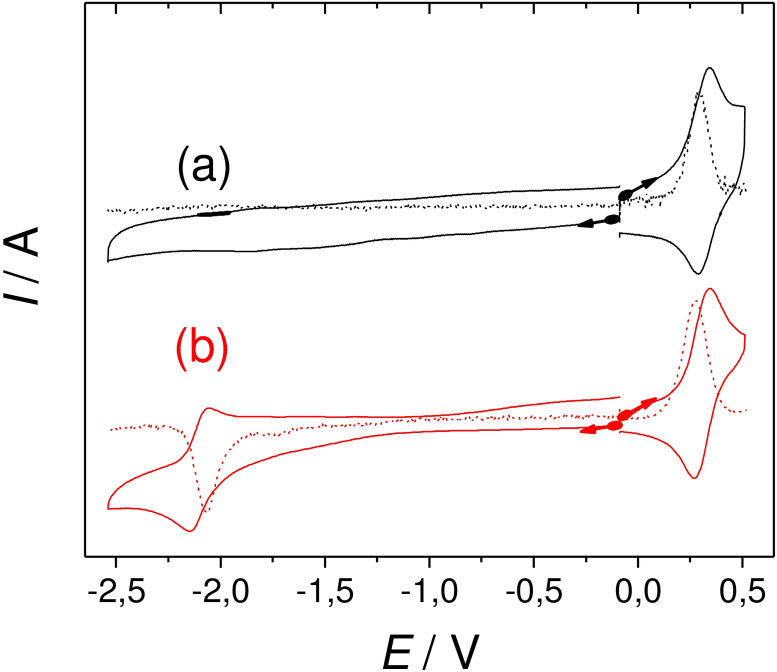
Cyclic voltammetry curves of (a) **[Gd(L_1_)]** and (b) **[Eu(L_1_)]** recorded in CH_3_CN (+0.1 M TBAP) at a carbon electrode in the glove box. Scan rate 0.1 V/s. In dotted lines: differential pulse voltammetry curves. Ref. Fc^+^/Fc.

The CV curves of the ligands are shown in Figure S11, while those of **[Gd(L_1_)]** and **[Gd(L_2_)]^+^** are depicted in Figure 2 (see Figure S12–13 for the other complexes). The potential values are listed in Table 1. The ligands show a reversible oxidation wave at E_1/2_^ox^=0.32 V. This value is close to that reported for the TEMPO^+^/TEMPO redox couple (E_1/2_^ox^=+0.25 vs Fc^+^/Fc in CH_3_CN)^[21]^ and hence assigned to the oxidation of the nitroxide moiety into an oxoammonium species. The ligands additionally display a very broad feature in the region −1.2 to −2 V vs. Fc^+^/Fc, which arises from the nitroxide/hydroxylamine redox couple (scheme 3). Both the assignment and an extraction of accurate potential values are however obscured by the broadening and the fact that dioxygen reduction also occurs in this potential range.^[21]^ Furthermore, the nitroxide/hydroxylamine redox system involves a proton‐couple‐to‐electron‐transfer (PCET), which is known to produce large peak separations.^[19]^

**Table 1:** Oxidation potentials of the complexes.^[a]^

Species	E_1/2_^ox^	Species	E_1/2_^ox^
**L_1_**	0.32	**L_2_**	0.32
**[Gd(L_1_)]**	0.34	**[Eu(L_1_)]**	0.34
**[Gd(L_2_)]^+^**	0.33	**[Eu(L_2_)]^+^**	0.33
**[Gd(L_3_)]^+^**	0.31	**[Eu(L_3_)]^+^**	0.33

[a] In 0.5 mM CH_3_CN solutions containing 0.1 M TBAP as supporting electrolyte. Note that a suspension might be observed in some instances due to the limited solubility in CH_3_CN. All the potentials values are given in V and referred to the Fc^+^/Fc redox couple. *T*=298 K; scan rate, 0.1 V/sec. Parameters for the ferrocene against the reference used (AgNO_3_ 0.01 M): E_1/2_=0.090 V.

The CV of the complexes show a well‐defined and reversible oxoammonium/nitroxide redox wave in the range +0.31 to +0.34 V (Table 1, Figure 2, Figure S12–13). These values fall within a sharp range for all the complexes, showing that both the presence of the metal and the formal charge of the complexes do not greatly influence the oxidation behaviour of these species. The reduction behaviour of **[Eu(L_1_)]** and **[Eu(L_2_)]^+^** differs from that of their gadolinium counterparts (Figure 2 and Figure S12): The first shows a reversible reduction wave at E_1/2_^red^ −2.10 V (ΔEp=0.10 V), while the second shows a quasi‐reversible reduction wave at E_1/2_^red^=‐1.01 V (ΔE_p_=0.45 V, Figure S13). Both reduction waves are assigned to the Eu(III)/Eu(II) redox system. The large peak separation in the second case attests that a chemical reaction or molecular rearrangement is associated to the electron transfer. The lower potential of **[Eu(L_1_)]** is in line with its neutral charge and the presence of three anionic donors, which better stabilize Eu(III) than the two carboxylate arms of **[Eu(L_3_)]^+^**. Surprisingly, no redox system associated with europium reduction could be clearly detected in the case of **[Eu(L_3_)]** ^+^. Note that a value of −1.10 V vs AgCl/Ag was reported for [Eu(DOTA)]^−^ in water at pH=7;^[22]^ The potential value of **[Eu(L_1_)]** is significantly lower, but direct comparison is difficult owing to the difference in solvent and possibly coordination sphere.

### Isotropic Electron Paramagnetic Resonance

**Figure 3:**
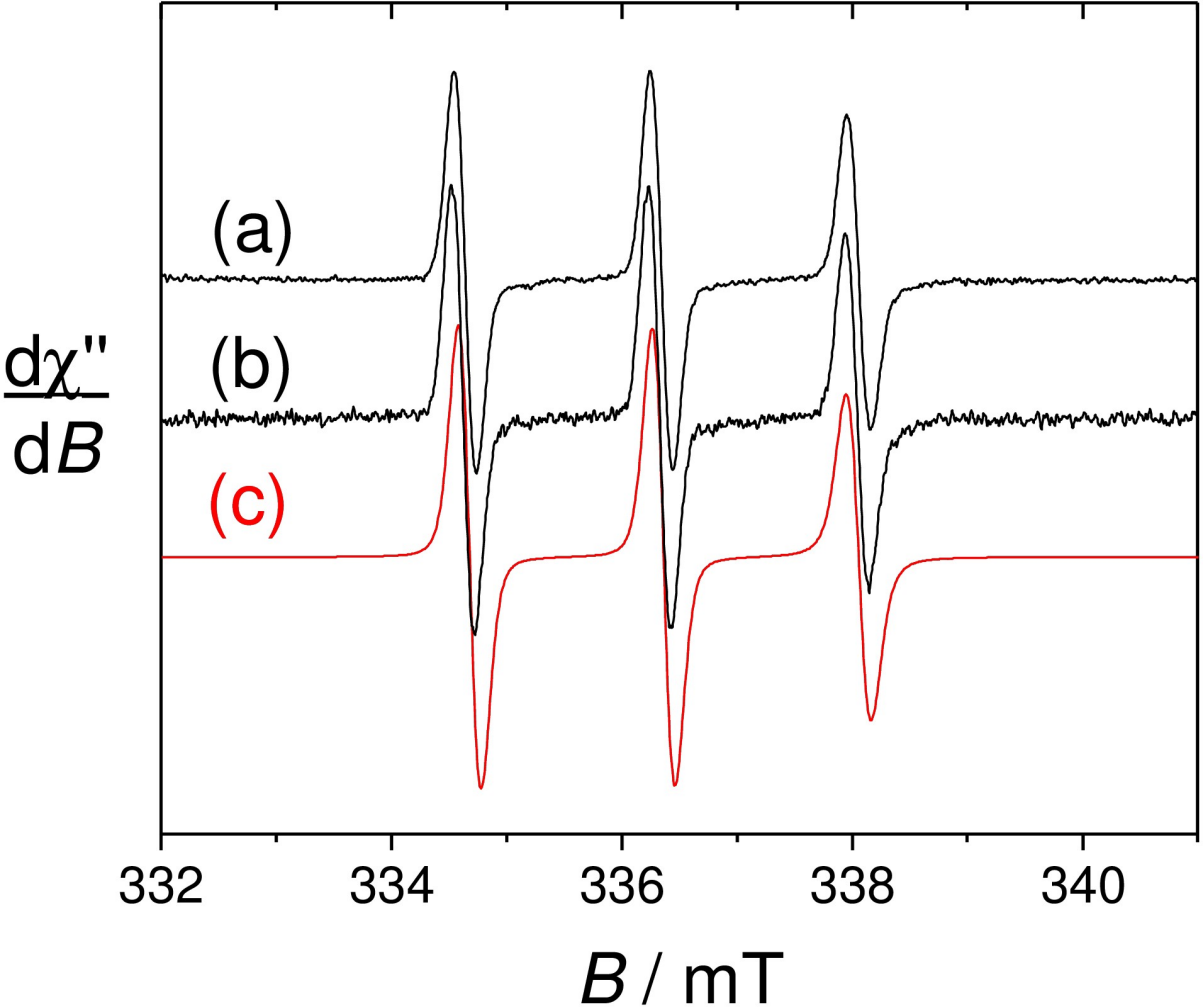
Isotropic EPR spectra of 0.5 mM solutions of (a) **(L_1_)**⋅ and (b) **(L_2_)**⋅ at pH 7 (0.1 M aqueous HEPES buffer). Red line: Simulation using the τ_c_ given in the text and an anisotropic A_N_ value corresponding to A_iso_=1.7 mT. Microwave Freq. 9.43 GHz, power. 3.5 mW; Mod. Amp. 0.2 mT, Freq. 100 KHz. *T*=293 K.

As stated in the synthesis section the ligands **L_1_** and **L_2_** were isolated in their diamagnetic form. Their nitroxide forms **(L_1_)**⋅ and **(L_2_)**⋅ were generated by dioxygen bubbling into an aqueous solution of the ligands at pH=9 (Figure 3a) followed by adjustment of the pH to 7 by addition of an HEPES buffer. The ligands show the three‐line pattern reminiscent of nitroxide radicals, whereby the ^14^N nuclear spin interacts with the electron spin. The A_N_ value is 1.7 mT and does not change significantly along the series due to the similar nature of the radical moiety. The relative heights of the high and low field resonances are not the same, which is due to the intrinsic anisotropy of the nitroxide radical. The relative ratio suggests fast tumbling of the molecules. Under this regime the rotational correlation times τ_c_, which is the time required for the molecule to rotate by 1 radian, could be calculated by using the Kivelson's formula (eq. 1), where C is a constant calculated from the principal values of g and A tensors of the nitroxide radical (6.6 10^−10^ for for di‐*tert*‐butylnitroxide), ΔH_(+1)_ is the peak‐to‐peak linewidth of the low field line and h_(+1)_ and h_(−1)_ are the height of the low and high field resonances.^[23]^

(1) $$\tau {}_{c}=C\;\Delta H{}_{\left(+1\right)}\left[\left(h{}_{\left(+1\right)}/h{}_{\left(-1\right)}\right){}^{1/2}-1\right]$$

The τ_c_ values are 1.7x10^−10^ sec for both **(L_1_)**⋅ and **(L_2_)**⋅, indicative of a high rotational freedom, in agreement with their low molecular weight. These values were confirmed by spectral simulation (Figure 3).

The europium complexes **[Eu(L_1_)]**, **[Eu(L_2_)]^+^** and **[Eu(L_3_)]^+^** are EPR‐silent, demonstrating ligand diamagnetism. Similarly to the ligands, a basic treatment produces the TEMPO radical species (**[Eu(L_1_)**⋅**]**, **[Eu(L_2_)**⋅**]^+^** and **[Eu(L_3_)**⋅**]^+^**), with their typical 3‐line pattern (Figure S14). The spectra of **[Eu(L_1_)**⋅**]** and **[Eu(L_2_)**⋅**]^+^** are very similar to those of the corresponding free ligands, both in terms of shape and intensity, due to the very weak magnetic moment of europium that does not alter the nitroxide signal. Noteworthy, the peak‐to‐peak linewidth is not strikingly different between **[Eu(L_3_)**⋅**]^+^** and **[Eu(L_1_)**⋅**]** (0.20 mT for both in the low field resonance), while no additional broad feature is detected in the EPR spectrum of the first. Hence despite of the presence of an octyl chain, **[Eu(L_3_)**⋅**]^+^** likely does not form micelles at the concentrations used for EPR measurements.^[24]^

**Figure 4:**
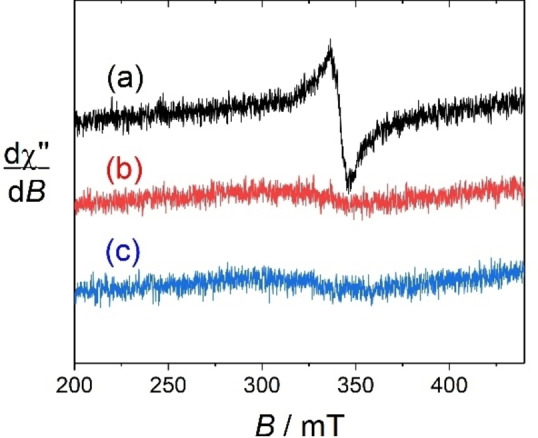
Isotropic EPR spectra of 0.5 mM solutions of (a) **[Gd(L_1_)]**, (b) **[Gd(L_2_)]^+^** and (c) **[Gd(L_3_)]^+^** at pH 6.8 (0.1 M aqueous HEPES buffer) obtained after addition of 4 molar equivalents of sodium ascorbate. Microwave Freq. 9.43 GHz, power. 7 mW; Mod. Amp. 0.4 mT, Freq. 100 KHz. *T*=293 K.

The gadolinium complex **[Gd(L_1_)]** displays a broad feature at g=1.98, with a peak‐to‐peak linewidth of 15 mT and no hyperfine splitting (Figure 4). This signal is assigned to the chelated Gd^3+^ ion.^[25]^ Indeed, the f sub‐shell is half‐field in Gd^3+^, resulting in a total electronic angular momentum (*L*=0). This makes the electronic relaxation much slower than that of the other paramagnetic trivalent lanthanides, for which the ligand‐field Hamiltonian has direct influence at the first order of perturbation,^[26]^ and the metal‐centered resonances can be observed even at room temperature.

The EPR spectra of the radical complex (generated using the same protocol than the europium complexes) **[Gd(L_1_)**⋅**]** demonstrates two sets of resonances (Figure S15). One is the sharp three‐line pattern (A_N_=1.7 mT; g=2.006, Γ=0.3 mT) that was already detected in the spectra of the free ligand **(L_1_)**⋅, which is consistently assigned to the nitroxide moiety. It must be stressed that the height of the nitroxide lines is significantly weaker in **[Gd(L_1_)**⋅**]** than in **(L_1_)**⋅. This is likely due to interactions with the highly paramagnetic (*S_Gd_* =7/2) center in the vicinity of the radical. The second signal is much broader (g=1.98, Γ=15 mT) and corresponds to the S‐state Gd^3+^ center (see above). The addition of ascorbate to an aqueous solution of **[Gd(L_1_)**⋅**]** at pH=6.8 (HEPES buffer) results in a quenching of the three‐line pattern, without affecting the gadolinium lines (Figure S15). This demonstrates that ascorbate reduces the nitroxide moiety into the diamagnetic hydroxylamine. The other gadolinium complexes **[Gd(L_2_)**⋅**]^+^** and **[Gd(L_3_)**⋅**]^+^** show similar nitroxide lines, but the gadolinium resonance proved to be more difficult to detect in the isotropic regime (Figure S16–17, Figure 4). We interpret this behaviour to distinct metal ion geometries (see below).

### Anisotropic Electron Paramagnetic Resonance

EPR at cryogenic temperatures can provide additional information regarding both Gd^3+^ centers difficult to detect at room temperature and magnetic couplings in complexes featuring two interacting paramagnetic centers. The spectrum of **[Gd(L_1_)**⋅**]** at 7 K shows a broad dissymmetrical resonance centered at g=2.00 (Γ=14 mT), which easily saturates, as classical for a Gd^3+^ signal (Figure S18).^[27]^ Additional less intense resonances can be observed at lower fields (g=6.5, 3.9) and attributed to satellite Gd^3+^ signals (Figure S19).^[28]^ At 21 K the broad dissymmetrical Gd^3+^ resonance is superimposed to a three‐line pattern reminiscent of the nitroxide radical. The plot of I as a function of 1/T (Figure S20) in the temperature range *T*=16–45 K mostly gave a line, suggesting that the coupling between the paramagnetic centers is weak.

**Figure 5:**
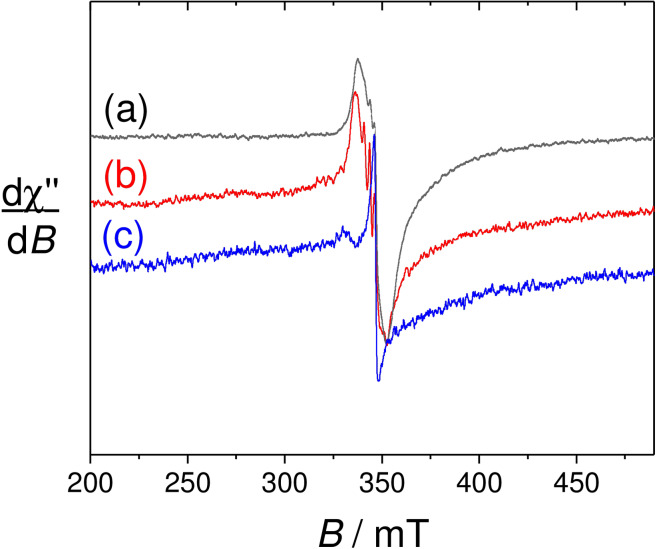
EPR spectra of 0.5 mM solutions of (a) **[Gd(L_1_)**⋅**]**, (b) **[Gd(L_2_)**⋅**]^+^** and (c) **[Gd(L_3_)**⋅**]^+^**. Microwave Freq. 9.63 GHz, power. Mod. Amp. Freq. 100 KHz. *T*=25 K.

The EPR spectrum of **[Gd(L_2_)**⋅**]^+^** shows the same global evolution, with gadolinium resonances at somewhat different g values (Figure 5, S21–22); broad pseudo axial resonances at g=2.5 and 2.00 and less intense resonances at lower fields (g=12.5, 5.8). The distinct gadolinium signature suggests a different environment or geometry of the metal in **[Gd(L_2_)**⋅**]^+^**, when compared to **[Gd(L_1_)**⋅**]**. For **[Gd(L_3_)**⋅**]^+^** the spectrum is again broad, with the most intense resonances at g=2.5 and 2.00 (Figure 5, S21,S23). These values compare well with to those measured for **[Gd(L_2_)**⋅**]**, suggesting no self‐aggregation. The nitroxide lines proved to be difficult to observe in this case, even at 40 K though they are clearly observed at room temperature.

### Luminescence Spectroscopy

**Figure 6:**
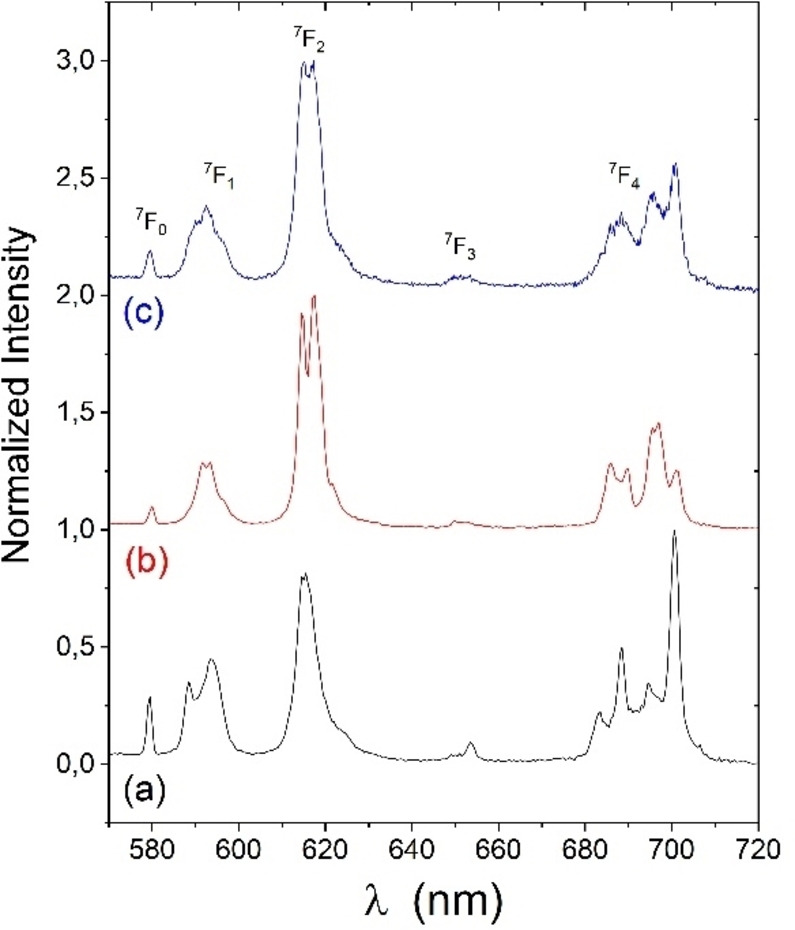
Luminescence spectra of the europium complexes: (a) **[Eu(L_1_)]**, (b) **[Eu(L_2_)]^+^** and (c) **[Eu(L_3_)]^+^** in H_2_O upon excitation at 395 nm. The ^5^D_0_→^7^F_x_ transitions are indicated.

We investigated the luminescence properties of the europium complexes, since this metal is particularly interesting for its emission in the visible region. The luminescence spectra of **[Eu(L_1_)]**, **[Eu(L_2_)]^+^** and **[Eu(L_3_)]^+^** are depicted in Figure 5. The complexes proved to be only very weakly luminescent due to the absence of a chromophore. Hence, we used a direct excitation into the metal centre at 396 nm. The excitation spectra taken from the emission band at 615 nm consists of the well‐defined spectrum of the metal centre, confirming excitation of the Eu(III) ion. The ratio of the bands in the **[Eu(L_1_)]** metal centred luminescence suggests symmetry of C2 or higher,^[29]^ with a strong ^7^F_0_ band at 580 nm and a reduced intensity of the ^7^F_2_ line (ca. 618 nm) compared to classic DOTA spectra (Figure 6).^[30]^ From luminescence spectra in H_2_O and D_2_O solutions and according to the Horrocks formula (Eq. 2)^[31]^ we calculated the number of coordinated water molecule q ∼1 for **[Eu(L_1_]**, which is consistent with an octadentate ligation by the macrocycle and completion of the coordination sphere to nine by a water molecule.

(2) $$q=1.11\times \left(1/\tau {}_{H2O}-1/\tau {}_{D2O}-0.31-0.45\;n{}_{\mathrm{NH}}-0.99\;n{}_{\mathrm{OH}}-0.075\;n{}_{\mathrm{CONHR}}\right)$$

$$\mathrm{Where}\;n{}_{\mathrm{NH}}=0,\;n{}_{\mathrm{OH}}=0,\;n{}_{\mathrm{CONHR}}=1$$

Luminescent spectra of **[Eu(L_2_)]^+^** were recorded to assess the difference in the coordination geometry due to the replacement of a carboxylate by an alkyne arm. The lifetime measurements upon direct excitation at 396 nm of **[Eu(L_2_)]^+^** gave values of τ=0.49 s and τ=1.29 s in water and deuterated water, respectively, giving q=1.1. This supports a heptadentate ligation by the ligand **L_2_** and the presence of one water molecules in the metal coordination sphere. This would imply an 8‐coordinate metal center, in contrast to **[Eu(L_1_)]**. Accordingly, the intensity ratio ^5^D_0_→^7^F_2_/^7^F_1_ is higher than in **[Eu(L_1_)]**, indicative of a distinct coordination geometry (Table 2). Eight‐coordinate europium ions are less common than nine‐coordinated ones in the coordination chemistry of DOTA, however examples have been reported for related macrocycles appended with phosphonate,^[32]^ mixed phosphonate and carboxylate,^[33]^ as well as pyridyl^[34]^ arms. Another possibility is the mixed coordination of water and an electrolyte present in solution (chloride) that would yield a nine‐coordinated metal center. We investigated this possibility by DFT calculations (see below). Given the low amount of chloride present in the solutions used for luminescence measurements (the only chloride source is the counter‐ion of **[Eu(L_2_)]^+^**) we consider such coordination unlikely. Furthermore, the r_1_ values of the gadolinium analogues also points towards one coordinated water molecule despite the use of a less coordinating triflate anion in this case (see below).

**Table 2:** Calculated q and intensity ratios from luminescence spectra.

Complex	q	Intensity ratio ^5^D_0_→^7^F_2_/^7^F_1_
**[Eu(L_1_)]**	0.9	1.7
**[Eu(L_2_)]^+^**	1.1	3.7
**[Eu(L_3_)]^+^**	0.7	3.1

Complex **[Eu(L_3_)]^+^** harbours an octyl chain, which was introduced via click chemistry. The so‐formed triazole group might be at an ideal distance for direct coordination. The lifetime measurements gave τ=0.57 s and τ=1.28 s in H_2_O and D_2_O, respectively, from which the number of coordinated water molecule was calculated to q=0.7. The intensity ratio ^5^D_0_→^7^F_2_/^7^F_1_ is 3.1, which is close to, but distinct from that measured for **[Eu(L_2_)]^+^**. We can therefore propose that one water molecule is present in the coordination sphere, with the triazole and one water molecule being present in the coordination sphere, which is confirmed by DFT calculations (see below).

It must be stressed that the redox‐active moiety is far from the coordination site (see below). We can thus safely assume that the number of coordinated molecules is unchanged between the hydroxylamine and the nitroxide (radical) form of the complexes.

### DFT calculations

**Figure 7:**
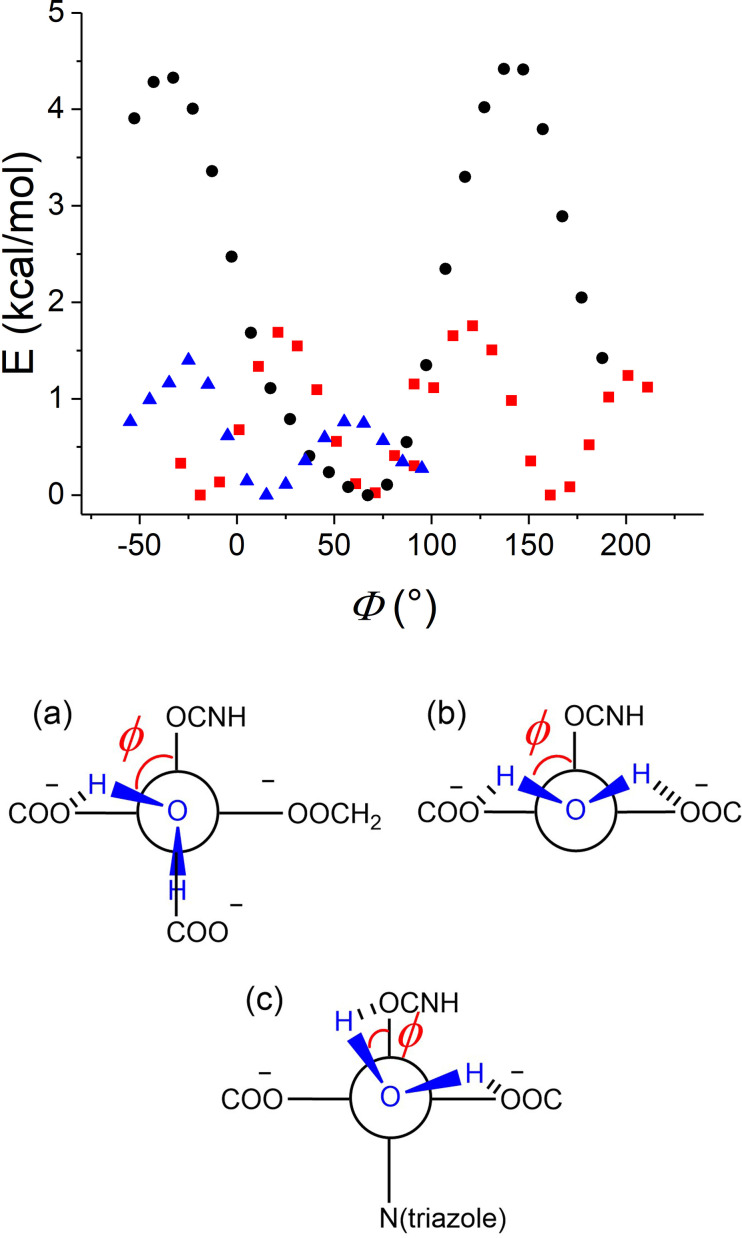
Potential energy scan (B3LYP/6‐31 g*(C,H,N,O)/ECP(Y)/PCM). *ϕ* is the dihedral angle between the O oxygen of the carbonyl), the metal ion, the O of the coordinated water molecule and one proton of this water molecule. Red squares: **[Y(L_1_)**⋅**]**; Black circles: **[Y(L_2_)**⋅**]** ^+^; Blue triangles: zoom for **[Y(L_3_)**⋅**]** ^+^. The lowest energy conformations are schematized: (a) **[Y(L_1_)**⋅**]**, (b) **[Y(L_2_)**⋅**]** ^+^ and (c) **[Y(L_3_)**⋅**]** ^+^.

The structure of the complexes has been investigated by DFT calculations using the number of coordinated water molecules determined by luminescence spectroscopy. In order to minimize the computational cost, the lanthanide ion has been replaced by Y^3+^, a metal commonly used as diamagnetic analogue of Gd^3+^. In the optimized structure the N−O function of the TEMPO unit points away from the metal, as previously observed for the chelate of **1** (Figure S24–26). In the case of **[Y(L_1_)**⋅**]** and **[Y(L_2_)**⋅**]^+^** (Figure S24–25) we conducted a relaxed potential energy scan (PES) by varying the dihedral angle H_w_−O_w_−Y−O(carbonyl), where H_w_ and O_w_ refers to the coordinated water molecule and O(carbonyl) the amide oxygen connecting the TEMPO unit to the cyclen (Figure 7). In the most stable conformation both H atoms of the water molecule are intramolecularly H‐bonded with two carboxylate oxygens of the macrocycle. They lie either in *cis* (**[Y(L_1_)**⋅**]**) (Figure 7a) or *trans* configuration (**[Y(L_2_)**⋅**]^+^**) (Figure 7b), with a much larger energy gap between the highest and lowest energy configurations in the second case. It is worth noting that in both cases the Y−O_w_ bond length varies inversely with the Y−O(carboxylate) bond distance along with the scan, with a longer Y−O_w_ bond distance in **[Y(L_1_)**⋅**]** in comparison to **[Y(L_2_)**⋅**]^+^**, due to the strong coordinating ability of the carboxylates, present in larger numbers. We also considered the coordination of a counter‐ion as a ninth ligand of **[Y(L_2_)**⋅**]^+^**. The structures with either chloride or triflate bound could be optimized (Figure S27), however thermochemistry shows that anion binding is not favoured (Table S1).

For **[Y(L_3_)**⋅**]^+^** we once again considered one coordinated water molecule, in agreement with luminescence data (Figure S26). We optimized geometries with the triazole group being either bound or uncoordinated. In the second case the H‐bonding network around the coordinated water molecule is the same than in **[Y(L_2_)**⋅**]^+^**. In the first case the coordinated water molecule is H‐bonded to one carboxylate oxygen and the carbonyl of the amide. A relaxed PES by varying the dihedral angle H_w_−O_w_−Y−O(carbonyl) confirmed that this conformation corresponds to an energy minimum. Thermochemistry calculations on the two geometry optimized structures (bound or unbound triazole, Figure S26) indicate that the structure corresponding to the coordinated triazole moiety is favoured by 4.9 kcal/mol (sum of electronic and thermal free energies). We conducted a second relaxed PES by varying the N(triazole) bond distance and starting from the optimized structure with the triazole bound: the lengthening of the N(triazole) bond results in a shortening of the Y−OH_2_ and Y‐carboxylate bonds, concomitant to an increase in energy (Figure S28). Together, these data support coordination of the triazole group in **[Y(L_3_)**⋅**]^+^**.

### Relaxivity

In order to determine the potential of the gadolinium complexes as MRI probes we performed fast field cycling NMR studies. The measurements were conducted in 2 mM buffered solutions of the gadolinium complexes under both their nitroxide and hydroxylamine forms. The first form is obtained by O_2_ bubbling at pH 9–10 and next addition of solid HEPES and pH adjustment until reaching a value of 7.4. The concentration of paramagnetic complex was controlled by using the Evans method.^[35]^ The reduced form was obtained by adding a slight excess of sodium ascorbate to the solution of the radical species.

**Figure 8:**
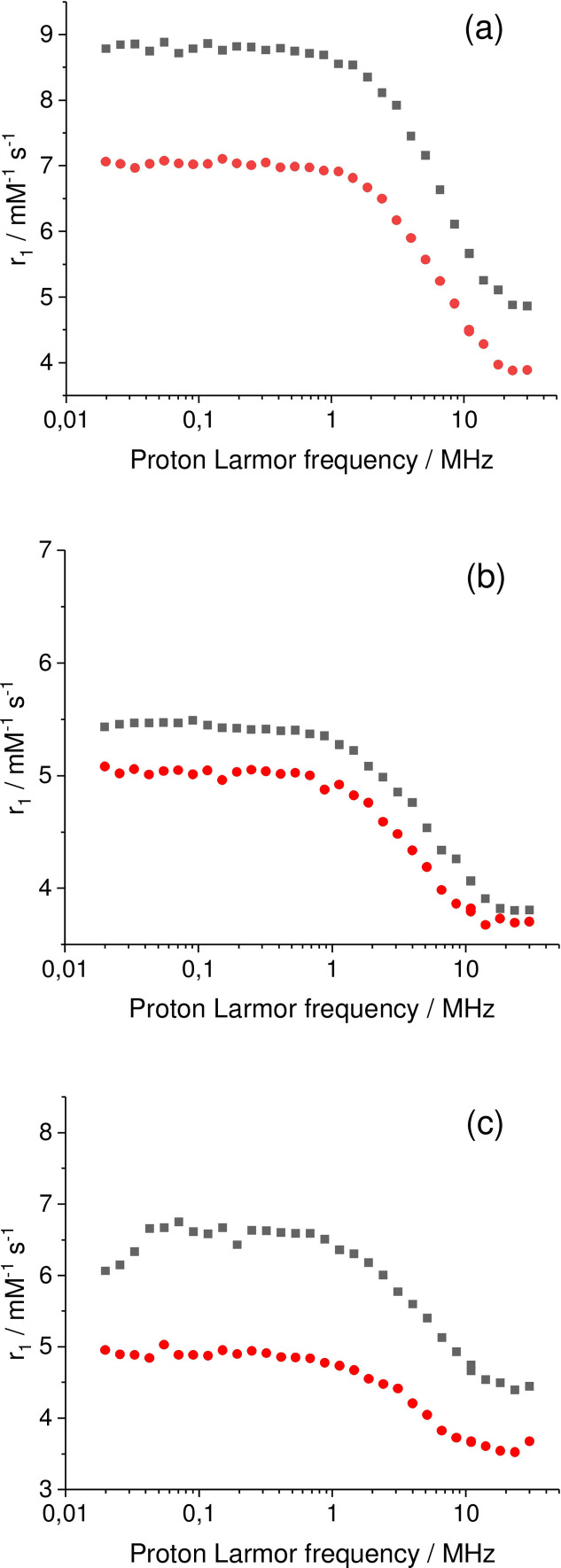
Relaxivity as a function of the frequency for 2 mM aqueous solutions (containing 0.1 M HEPES, pH 7.4) of: a) **[Gd(L_1_)**⋅**]**; b) **[Gd(L_2_)**⋅**]^+^**; c) **[Gd(L_3_)**⋅**]^+^**. Black curve: nitroxide form. Blue curve: hydroxylamine form (generated upon addition of two equivalents of sodium ascorbate to the radical). *T*=298 K.

The evolution of the relaxivity as a function of the frequency at 298 K is depicted in Figure 8. The *r*_1_ values for the nitroxide complexes **[Gd(L_1_)**⋅**]**, **[Gd(L_2_)**⋅**]^+^** and **[Gd(L_3_)**⋅**]^+^** at 30 MHz are summarized in Table 3 together with the values for the hydroxylamine derivatives **[Gd(L_1_)]**, **[Gd(L_2_)]^+^** and **[Gd(L_3_)]^+^** species. The *r*_1_ value of **[Gd(L_1_)]** at 30 MHz is 3.8 mM^−1^ s^−1^, which is slightly smaller than [Gd(DOTA)(H_2_O)]^−^ (4.7 mM^−1^ s^−1^),^[36]^ but yet consistent with a single water molecule in the inner coordination sphere of the metal ion. This q number is consistent with that determined by luminescence on **[Eu(L_1_)]**. Both the europium and gadolinium complexes can indeed be considered isostructural, making luminescence and relaxivity complementary techniques for the determination of q values.^[33]^ The *r_1_* value is within the same range for both **[Gd(L_2_)]^+^** and **[Gd(L_3_)]^+^** (3.7 mM^−1^ s^−1^), again demonstrating one water molecule in the coordination sphere. All these *r_1_* values are fully consistent with luminescence measurements on the europium derivatives. A remarkable trend is observed when the nitroxide form is compared to the hydroxylamine one, whereby the *r_1_* value is higher. The magnitude of this effect is larger at lower frequency. It is also larger for **[Gd(L_1_)]** than for the other complexes whatever is the frequency. At 30 KHz the increase in relaxivity upon oxidation is for instance 26%, 8% and 22% for **[Gd(L_1_)]**, **[Gd(L_2_)]^+^** and **[Gd(L_3_)]^+^**, respectively. This increase can be understood by the generation of a paramagnetic center in the vicinity of the gadolinium ion which accelerates spin relaxation. It is worth noting that **[Gd(L_3_)]^+^** (and its radical counterpart) displays *r_1_* values in the same range than **[Gd(L_2_)]^+^**. The value is much smaller than those reported for DOTA derivatives with micellar properties. As an example, the gadolinium complex of the 1,4,7,10‐tetraazacyclododecane‐1‐[1′‐carboxy‐1′‐dodecyl(methyl)amino‐oxoethyl]‐4,7, 10‐triacetic acid features one coordinated water molecule and assembles as micelles at pH=7.4, giving 18.0 mM^−1^ s^−1^ at 20 MHz.^[37]^ This further shows that high molecular weight assemblies (e. g. micelles) likely do not form (or hardly form) under our experimental conditions, despite the small increase in relaxivity at high frequency (30 MHz).

**Table 3:** Relaxivity of the complexes.^[a]^

Complex	*r_1_*/mM^−1^ s^−1^ (30 KHz, 0.7 T)	*r_1_*/mM^−1^ s^−1^ (30 MHz, 0.7 T)
**[Gd(L_1_)]**	7.0	3.8
**[Gd(L_2_)]^+^**	5.1	3.7
**[Gd(L_3_)]^+^**	5.0	3.7
**[Gd(L_1_)**⋅**]**	8.8	4.8
**[Gd(L_2_)**⋅**]^+^**	5.5	3.8
**[Gd(L_3_)**⋅**]^+^**	6.1	4.4

[a] pH=7.4 in 2 mM aqueous HEPES buffer (0.1 M) at 298 K.

### Biological studies

**Figure 9:**
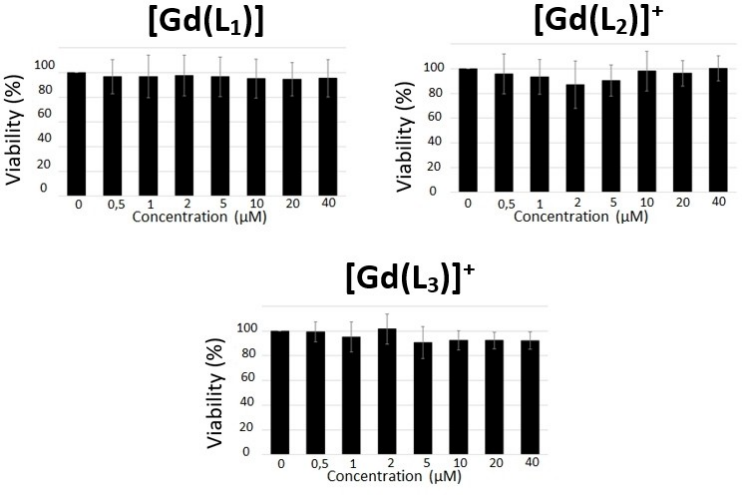
Cellular viability of M21 cells 72 h after treatment with increasing amount of gadolinium complexes (from MTT assays).

It is documented that the nitroxide/hydroxylamine redox potential lies within the biologically relevant potential range for monitoring *in vivo* redox events.^[17b]^ Hence the three probes were expected to give interesting information on the redox status in the environment. With this in mind the complexes were tested for their cytotoxicity in a M21 cell line. The complexes were tested under their isolated hydroxylamine form in the concentration range 0.5–40 μM, while the viability was measured up to 72 h. Note that the oxidation state complexes may evolve during this long period since the hydroxylamine is prone to oxidation by air and the nitroxide prone to reduction by biological reductants. Overall, MTT assays demonstrate no toxicity of the gadolinium complexes **[Gd(L_1_)]**, **[Gd(L_2_)]^+^** and **[Gd(L_3_)]^+^** over a 72 hours period at concentrations as high as 40 μM (Figure 9, S29).

**Figure 10:**
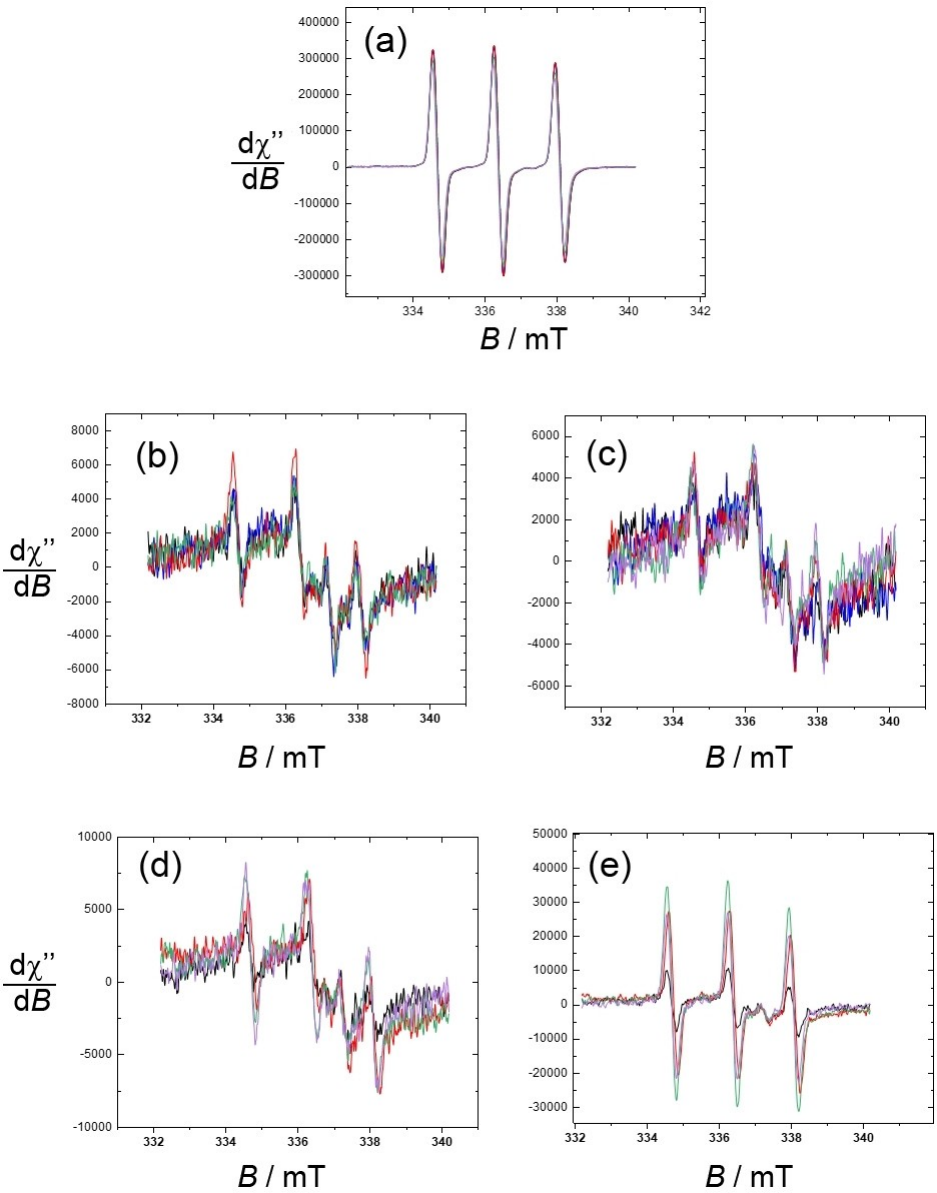
Isotropic EPR spectra of cells incubated with a 200 μM solution of **[Eu(L_2_)**⋅**]^+^**. a) Supernatant; b) cell pellets immediately after incubation; c) cell pellets after incubation and further 24 h under air; d) lysate immediately after incubation; e) lysate after incubation and further 24 h under air. Incubation times: black) 0 min; blue) 5 min; red) 10 min; green) 30 min; purple) 60 min. *T*=298 K. Microwave Freq. 9.43 GHz, power. 17.8 mW; Mod. Amp. 0.35 mT; Mod. Freq. 100 KHz. *T*=293 K.

In order to determine if the complexes penetrated the cell membrane a series of controls were carried out where EPR measurements were performed on the centrifuged whole cells, the supernatant and a cell lysate after a short treatment (less than 1 h) with the complexes. The radical form of the complexes had to be used since it is the only one that can be detected by EPR (through the nitroxide resonances) at concentrations of 200 μM. Solutions of the complexes diluted in the culture medium were used as blanks. Both the gadolinium and europium complexes were investigated for cellular penetration (Figure 10, S30), the second allowing for a straightforward detection. EPR spectra performed on the supernatant demonstrated a 3‐line nitroxide signal of intensity comparable to that of the same complex in a culture medium. Hence, within the accuracy of EPR the complexes remain in the culture medium. The codetection of the gadolinium resonance in the case of **[Gd(L_1_)**⋅**]^+^** confirms the integrity of the complex in the cell medium. Both the cells after centrifugation or cell lysates demonstrated no nitroxide signal for all the complexes, attesting that they do not penetrate cells, with the exception of **[Eu(L_2_)**⋅**]^+^**. In this particular case, a very weak 3‐line pattern of the nitroxide was observed. In order to confirm this result, we increased the number of cells from 2×10^5^ to 8×10^5^ and performed the same set of experiments. The intensity of the 3‐line nitroxide signal of the supernatant slightly decreases with time (14% after 1 h). Consistently, the nitroxide resonances could be detected in whole cells after centrifugation or cell lysates with a higher intensity in comparison to the first set of experiment. The cells after centrifugation and cell lysates were next allowed to stand for 24 h at room temperature under air and the EPR spectra were recorded again. The cell pellets demonstrated no change, but a dramatic increase (6 times) was observed for cell lysates. We hypothesize that the complex was internalized into the cells, resulting in a quenching of the nitroxide function by biological reductants (glutathione, thiols for example). After lysis, the biological material is exposed to dioxygen, resulting in a re‐oxidation of the hydroxylamine, producing the characteristic 3‐line pattern. From the maximum EPR intensity in the cell lysate we calculated that ca. 10% of **[Eu(L_2_)**⋅**]^+^** has penetrated the cells, which corresponds to a concentration of 20 μM. It is surprising that the derivatives of **L_3_** do not enter the cells. This result might be rationalized by the fact that the alkyl chain is too short to promote micelle formation or interactions that would improve diffusion through the bilayer.

## Conclusions

In conclusion three redox active ligands have been developed and their complexation with lanthanide salts performed. The complexes feature one nitroxide moiety which can shuttle between paramagnetic and diamagnetic oxidation states. They all exhibit one coordinated water molecule as determined by luminescence measurements on the europium complexes (the structures were confirmed by DFT calculations), making the gadolinium derivatives suitable candidates for MRI imaging. Relaxivity measurements demonstrated a redox state dependent magnetic response which was characterised by an increase in *r_1_* upon radical formation. It is significant that the alkyne group provides a simple way for post‐functionalization through click reaction, hence offering large opportunities for future optimization. Finally, the complexes proved to be not toxic at concentrations as high as 40 μM, which is a prerequisite for biological applications.

## Experimental Section

### Materials and Methods

All chemicals were of reagent grade and were used without purification. NMR spectra were recorded on a Bruker AM 400 (^1^H at 400 MHz) spectrometer. Chemical shifts are quoted relative to tetramethylsilane (TMS). Mass spectra were recorded on an ESI/QTOF Waters Xevo G2‐S apparatus. The FTIR spectra were recorded using a Nicolet iS10 spectrometer on crystalline material (ATR mode). UV/Vis spectra were recorded on a Cary Varian 50 spectrophotometer equipped with a Hellma immersion probe (1.000 cm path length). The temperature in the cell was controlled using a Lauda circulating bath. Luminescence data were recorded both at room and low temperature using a modular Fluorolog FL3‐22 spectrometer from Horiba‐Jobin Yvon‐Spex equipped with a double grating excitation monochromator and an iHR320 imaging spectrometer. Hamamatsu R928P and R5509 photomultipliers were used for visible and NIR measurements, respectively. All spectra were corrected for detection and optical spectral response (instrumental functions) of the spectrofluorimeters. X‐band EPR spectra were recorded on a Bruker EMX Plus spectrometer equipped with a Bruker Helium flow cryostat and a dual mode cavity. Electrochemical measurements were carried out using a CHI 620 potentiostat. Experiments were performed in a standard three‐electrode cell under argon atmosphere. A glassy carbon disc electrode (3 mm diameter), which was polished with 1 mm diamond paste, was used as the working electrode. The auxiliary electrode is a platinum wire, while the reference was an Ag/AgNO_3_ 0.01 M in CH_3_CN. All the potentials are given vs. the Fc^+^/Fc redox couple, which was used as standard. Note that a suspension might be observed in some instances due to the limited solubility of the compound in CH_3_CN.

The relaxivity values *r*_1_ were derived from the experimental longitudinal relaxation times *T*_1_ of the water protons.^[38]^ The *T*_1_ values were measured from 20 kHz to 30 MHz with a commercial Spinmaster FFC 2000 Stelar relaxometer^[39]^ (Stelar s.r.l., Mede PV, Italy). The prepolarized (PP) and non‐polarized (NP) sequences (see Figure 1 of Ref.^[39a]^) were used below and above about 12 MHz, respectively. A high polarization field *B*_pol_ corresponding to a proton resonance frequency of 28 MHz was employed in the PP experiments. For each magnetic field r_1_ was calculated according to r_1_=(R_1_‐R_10_)/[complex], where R_1_ is the relaxation rate measured in the presence of the complex, R_10_ the relaxation rate measured in the absence of complex (typically 0.4 s^−1^). The complex concentration is expressed in mM.

### DFT Calculations

Full geometric optimizations were performed with the Gaussian 9.0 program.^[40]^ The B3LYP functional^[41]^ was used together with the 6‐31 g* basis set for the C,H,N atoms^[42]^ and a pseudo potential (LanL2DZ)^[43]^ for the central metal ion, by using a polarized continuum model for the solvent. Frequency calculations were systematically performed on the optimized structures in order to ensure that they correspond to a real energy minimum and not a saddle point. The relaxed potential energy surface scans were performed by varying dihedral angles and/or bond distances.

### Cell culture

Human melanoma cell line M21 was purchased from ATCC (Manassas, VA, USA). Cells were cultured in DMEM medium supplemented with 10% (v/v) fetal calf serum (FCS) and 2 mM glutamine (Thermo Fisher Scientific, Courtaboeuf, France). Cells were maintained at 37 °C in a 5% CO_2_‐ humidified atmosphere and tested to ensure freedom from mycoplasma contamination. All cell lines were used within 5–50 passages of thawing the original stocks.

### MTT assay

M21 cells were seeded into 96‐well plates (1.5×10^3^ cells per well) in 100 μl of culture medium. After 24 h, cells were treated with the lanthanide(III) complexes at various concentrations. Following incubation for 24 h, 48 h or 72 h, 10 μl of a MTT (3‐(4,5‐dimethylthiazol‐2‐yl)‐2,5‐diphenyltetrazolium bromide) stock solution (Euromedex, Mundolsheim, France) in PBS at 5 mg ml^−1^ was added in each well and the plates were incubated at 37 °C for 2 h. To solubilize water‐insoluble purple formazan crystals, SDS 10%/HCl 0.1% solution was used. After 24 h, absorbance was measured on an ELISA reader (Tecan, Lyon, France) at a test wavelength of 570 nm and a reference wavelength of 650 nm.

### Cellular penetration by EPR spectroscopy

M21 cells were seeded in 4‐well plates (1×10^5^ cells per well) in 1 mL of culture medium. After 24 h, cells were incubated with the complexes at 200 μM concentration in 200 μL during 5 min, 10 min, 30 min or 1 h. Cell culture medium was recovered and M21 cells where harvested. A fraction of M21 pellets was then washed twice in cold PBS and incubated in lysis buffer (10 mmol/L Tris–HCl pH 7.5, 120 mmol/L NaCl, 1 mmol/L EDTA, 1 mmol/L dithiothreitol, 0.5% Nonidet P‐40, 0.05% sodium dodecyl sulfate), supplemented with protease inhibitors (Na_3_VO_4_ and NaF). After 10 min on ice lysates were centrifugated at 20,000×g for 15 minutes, and soluble fraction was recovered. Cell culture medium, pellets of whole M21 cells or M21 cell lysates were then analyzed by EPR spectroscopy.

### Synthesis

Intermediate **2** (1,4,7‐bis(*tert*‐butoxycarbonylmethyl)‐4‐[2’,2’,6’,6,’‐tetramethyl‐1’‐oxyl‐4’‐piperidyl)amide]‐1,4,7,10‐tetrazacyclododec‐1‐yl]acetate, free radical). Potassium carbonate (310 mg, 2.25 mmol) and potassium iodide (124 mg, 0.748 mmol) were added to a solution of DO_3_A^t^Bu (385 mg, 0.748 mmol) in MeCN (50 mL) and the suspension was stirred for 10 minutes. 4‐(2‐chloroacetamido)‐2’,2’,6’,6,’‐tetramethylpiperidine‐1’‐oxyl^[17a]^ (186 mg, 0.748 mmol) was then added to the reaction and the resulting mixture was refluxed overnight. Completion of the reaction was monitored by TLC (MeOH 1: 9 CH_2_Cl_2_) and inorganic residuals were filtered off. Solvent was removed by evaporation then the residue was dissolved in NaOH (3 M) and extracted with CH_2_Cl_2_ (3×40 mL). Organic phases were combined, dried over Na_2_SO_4_ and evaporated under reduced pressure to yield the crude product as a brownish oil. Purification by DCVC (gradient from 0 up to 20% MeOH in CH_2_Cl_2_) yielded after evaporation the pure product (**2**) as a brown oil (510 mg, 0.702 mmol, 94%). HRMS‐ESI: *m/z* calc. for C_37_H_69_O_8_N_6_Na ([M+Na]^+^), 748.5069; found, 748.5055. EPR (MeCN): Isotropic TEMPO nitroxide 3‐line pattern signal at g=2.01, A_N_=1.7 mT.

**L_1_** (1,4,7‐tris(carboxymethyl)‐10‐[2‐[(1‐hydroxy‐2,2,6,6‐tetramethyl‐4‐piperidyl)amino]‐2‐oxo‐ethyl]‐1,4,7,10‐tetrazacyclododec‐1‐yl]acetic acid). A mixture of trifluoroacetic acid (5 mL) and CH_2_Cl_2_ (20 mL) was added to (2) (510 mg, 0.702 mmol). The brown solution immediately turned intense purple. The reaction mixture was stirred overnight at room temperature and evaporated under reduced pressure. The residue was retaken in CH_2_Cl_2_ (4×75 mL), MeOH (3×75 mL) and evaporated again to remove all traces of acid. The residue was solubilized in a minimum of methanol (MeOH) and precipitated by addition of diethylether (Et_2_O). The yellow solid was filtered off, washed with Et_2_O and dried under vacuum to obtain a product **L_1_** as a yellow powder (291 mg, 74%). ^1^H NMR (500 MHz, D_2_O) δ=4.27 (m, 1H, N‐CH TEMPOH ring), 3.90–3.81 (br, 4H, 2x N‐CH_2_ acetate), 3.66 (s, 2H, N‐CH_2_ amide), 3.48 (br, 2H, N‐CH_2_ acetate), 3.46–3.37 (br, 8H, NCH_2 cycle_n), 3.24–3.07 (br, 8H, N‐CH_2 cycle_n), 2.25 (d, 14 Hz 2H, CH_2_ tempo ring), 1.81 (t, 14 Hz, 2H, CH_2_ tempo ring), 1.51 (s, 6H, CH_3_ TEMPOH ring), 1.46 (s, 6H, CH_3_ TEMPOH ring). ^13^C NMR (125 MHz, D_2_O) δ=180.0, 173.2, 171.0, 163.4, 163.1, 162.8, 117.5, 115.2, 61.0, 56.6, 51.1, 48.3, 40.7, 30.2, 30.0, 27.4, 19.5. IR (cm^−1^, neat): 3379, 3252, 3079, 2971, 2822, 1679, 1586, 1404, 1328, 1286, 1245, 1203, 1175, 1122, 1105, 995, 969, 926, 897, 799, 717. HRMS‐ESI: *m/z* calc. for C_25_H_45_O_8_N_6_ ([M+H]^+^), 557.3304; found, 557.3295

Intermediate **3** (1,7‐bis(*tert*‐butoxycarbonylmethyl)‐4‐prop‐2‐ynyl‐1,4,7,10‐tetreazacyclododecane). To a solution of DO2AtBu (1.724 g, 4.30 mmol)^[44]^ and potassium carbonate (0.53 g, 3.44 mmol) in MeCN (150 mL) was added propargyl bromide (0.26 mL, 4.30 mmol) in MeCN (100 mL) dropwise at −30 °C in an acetone/liquid nitrogen bath over 30 min. The reaction mixture was then allowed to warm at room temperature and stirred for 48 hours. Inorganic residuals were removed by filtration and solvent was removed under reduced pressure. The resulting yellow solid was dissolved in NaOH (3 M) and was extracted with CHCl_3_ (3×80 mL). Organic phases were combined, dried over Na_2_SO_4_ and evaporated under reduced pressure to yield the crude product. The product was then purified over a small amount of silica gel by eluting with EtOAc to remove impurities and then MeOH/NH_4_OH (7 : 3 v/v) to remove the product from the silica gel. Traces of silica were not observed even after solvent removal due to the small solvent volume used. The solvent was evaporated to afford 1,7‐bis(*t*‐butoxycarbonylmethyl)‐4‐alkylmethyl‐1,4,7,10‐tetreazacyclododecane (**3**) as a yellow oil corresponding to the pure product (1.53 g, 81% yield). ^1^H NMR (500 MHz, CDCl_3_) δ=3.44 (s, 2H, NCH_2_‐alkyne), 3.27–3.32 (4H, NCH_2_CO^t^Bu), 2.59–2.83 (16H, NCH_2 cycle_n), 2.13 (s, 1H, CH‐alkyne), 1.45 (s, 18H, CH_3_‐^t^Bu). ^13^C NMR (100 MHz, CDCl_3_) δ=173.01, 171.25, 81.77, 80.94, 80.78, 72.54, 57.86, 57.59, 57.01, 55.73, 52.05, 51.76, 50.73, 47.94, 45.72, 28.23. IR (cm^−1^, neat): 3281, 2975, 2933, 2821, 2359, 1725, 1455, 1394, 1367, 1256, 1217, 1149, 1118, 1072, 935. HRMS‐ESI: *m/z* calc. for C_23_H_43_O_4_N_4_ ([M+H]^+^), 439.3279; found, 439.3272.

Intermediate **4** (1,7‐bis(t‐butoxycarbonylmethyl)‐4‐[2’,2’,6’,6,’‐tetramethyl‐1’‐oxyl‐4’‐piperidyl)amide]‐10‐prop‐2‐ynyl‐1,4,7,10 tetrazacyclododec‐1‐yl]acetate). To a solution of (**3**) (404 mg, 0.92 mmol) in MeCN (140 mL) was added potassium carbonate (509 mg, 3.68 mmol) and potassium iodide (168 mg, 1.01 mmol) and the suspension was stirred for 10 minutes. 4‐(2‐chloro‐acetamido)‐2,2,6,6‐tetramethylpiperidine‐1‐oxyl (251 mg, 1.01 mmol) was then added to the reaction and the resulting mixture was refluxed 4 hours and stirred overnight at room temperature. Completion of the reaction was monitored by TLC (EtOAc) and inorganic residuals were filtered off. Solvent was removed by evaporation and the crude product was purified over a small pad of silica gel by eluting with EtOAc. Solvent was evaporated under reduced pressure to yield pure compound (**4**) as a brownish oil (598 mg, quantitative). IR (cm^−1^, neat): 3224, 2978, 2934, 2864, 2832, 1728, 1669, 1543, 1456, 1394, 1368, 1314, 1227, 1155, 1107, 908, 851. HRMS‐ESI: *m/z* calc. for C_34_H_62_O_6_N_6_ ([M+H]^+^), 650.4725; found, 650.4730. EPR (MeCN): Isotropic TEMPO nitroxide 3‐line pattern signal at g=2.01, A_N_=1.7 mT.

**L_2_** (1,7‐bis‐(carboxymethyl)‐4‐[2‐[(1‐hydroxy‐2,2,6,6‐tetramethyl‐4‐piperidyl)amino]‐2‐oxo‐ethyl]‐10‐prop‐2‐ynyl‐1,4,7,10‐tetrazacyclododec‐1‐yl]acetic acid). Compound (**4**) (598 mg, 0.92 mmol) was solubilized in a mixture of trifluroacetic acid and CH_2_Cl_2_ (50 mL, 1 : 4 v/v). The brown solution immediately turned intense purple. The reaction mixture was stirred overnight at room temperature and concentrated under reduced pressure. The residue was dissolved in CH_2_Cl_2_ (4×75 mL), MeOH (3×75 mL) and evaporated again to remove all traces of acid. The residue was solubilized in a minimum of MeOH and precipitated by addition of Et_2_O. The brownish solid was filtered off, washed with Et_2_O and dried under vacuum. The crude product was then purified by recrystallization in a minimum of ethanol to afford the desired compound (L_2_) as a brown solid (431 mg, 87%). ^1^H NMR (500 MHz, CDCl_3_) δ=4.32–4.27 (m, 1H, NCH‐tempo ring), 3.93–3.84 (m, 4H, NCH_2_CO_2_H) 3.69 (2H, NCH_2_CCH), 3.58–3.38 (br, 8H, NCH_2 cycle_n), 3.16–3.06 (br, 8H, NCH_2 cycle_n), 2.68 (s, 1H, CH‐alkyne), 2.25 (t, 14 Hz, 2H, CH_2_‐TEMPO), 1.81 (t, 13 Hz, 2H, CH_2_‐TEMPO), 1.52 (s, 6H, CH_3_‐TEMPO), 1.45 (s, 6H, CH_3_‐TEMPO). ^13^C NMR (125 MHz, CDCl_3_) δ=173.01, 171.25, 81.77, 80.94, 80.78, 72.54, 57.86, 57.59, 57.01, 55.73, 52.05, 51.76, 50.73, 47.94, 45.72, 28.23. IR (cm^−1^, neat): 3281, 2975, 2933, 2821, 2359, 1725, 1455, 1394, 1367, 1256, 1217, 1149, 1118, 1072, 935. (HRMS‐ESI): *m/z* calc for C_23_H_43_O_4_N_4_ ([M+H]^+^), 539.3552; found, 539.3533.

**General procedure for the complexation**: the appropriate Ln^3+^ salt (0.10 mmol) and the ligand (0.10 mmol) were dissolved in distilled water (25 mL). The pH of the solution was adjusted to 7.0 by addition of NaOH (0.5 M) until the pH was stable. The solution was then heated to 60 °C overnight. Water was then removed under reduced pressure. The residue was dissolved in a minimum amount of MeOH and precipitated by addition of Et_2_O. The precipitate was decanted, solvents were removed and after 3 rinsing with Et_2_O, the product was isolated as a bright yellow powder.

**[Gd(L_1_)]**. From **L_1_** (75 mg, 0.140 mmol) and gadolinium triflate (84 mg, 0.140 mmol). Yield 99 mg (84%). HRMS‐ESI: *m/z* calcd for C_25_H_42_GdN_6_O_8_Na ([M+H]^+^), 735.2199; found, 735.2194. IR (cm^−1^, neat): 713.9, 800.6, 833.5, 902.3, 938.1, 1000.9, 1027.8, 1078.7, 1159.4, 1237.2, 1276.0, 1314.9, 1362.7, 1383.7, 1464.4, 1589.4, 2863.4, 2923.6, 2974.5, 3321.6, 3415.2.

**[Eu(L_1_)]**. From **L_1_** (30 mg, 0.056 mmol) and europium chloride (14.5 mg, 0.056 mmol). Yield 35 mg (86%). HRMS‐ESI: *m/z* calcd for C_25_H_43_EuN_6_O_8_ ([M+H]^+^); 706.2335 found, 706.2331. IR (cm^−1^, neat): 713.9, 839.5, 896.3, 932.1, 998.0, 1084.7, 1153.4, 1237.2, 1311.9, 1362.7, 1386.4, 1458.4, 1597.07, 2860.9, 2926.6, 2980.5, 3243.6, 3415.0.

**[Gd(L_2_)](OTf)**. From **L_2_** (75 mg, 0.140 mmol) and gadolinium triflate (84 mg, 0.140 mmol). Yield 99 mg (84%). HRMS‐ESI: *m/z* calcd for C_26_H_43_O_6_N_6_Gd ([M+H]^+^), 693.24857; found, 693.24824. IR (cm^−1^, neat): 638.0, 713.6, 791.4, 836.2, 928.9, 961.8, 1030.7, 1078.4, 1170.4, 1207.7, 1252.5, 1394.4, 1518.0, 1601.8, 1679.5, 2866.6, 2977.2, 3111.8, 3276.3, 3473.6.

**[Eu(L_2_)](Cl)**. From **L_2_** (30 mg, 0.056 mmol) and europium chloride (14.5 mg, 0.056 mmol). Yield 35 mg (86%). HRMS‐ESI: *m/z* calcd for C_26_H_43_O_6_N_6_Eu ([M+H]^+^), 689.25312; found, 689.25145. IR (cm^−1^, neat): 713.5, 795.8, 836.9, 925.6, 969.9, 1001.6, 1082.5, 1130.8, 1199.8, 1318.2, 1375.2, 1435.4, 1589.0, 1653.2, 1676.7, 2872.8, 2983.6, 3085.0, 3240.1, 3373.1.

**Procedure for the synthesis of [Ln(L_3_)]^+^**: 1‐azido‐octane (1.5 eq) was added to a solution of **[Ln(L_2_)]^+^** (1.0 eq) in DMF (5 mL) with sodium ascorbate (1.2 eq) to ensure the complete reduction of the nitroxide. After 5 minutes, a solution of copper (II) sulfate pentahydrate (0.1 eq) with sodium ascorbate (1.2 eq) in water (5 mL) was added to the reaction. In this case, sodium ascorbate was used to generate the catalyst Cu (I). The reaction was stirred for 3 days at 30 °C and the completion of the reaction was followed by mass spectrometry (ESI). Solvents were then removed by evaporation under reduced pressure, the residues were dissolved in a minimum amount of MeOH and addition of Et_2_O initiated the precipitation of a white powder. After decantation by centrifugation, the supernatant was removed with a Pasteur pipette and after drying, **[Ln(L_3_)]^+^** was obtained as a white powder.

**[Gd(L_3_)](OTf)**. From **[Gd(L_2_)]**(OTf) (30 mg, 36 μmol, 1 eq) with sodium ascorbate (8.5 mg, 43 μmol, 1.2 eq), octane‐azide (10 mg, 54 μmol, 1.5 eq) and copper sulfate pentahydrate (0.90 mg, 3.6 μmol, 0.1 eq) with sodium ascorbate (8.5 mg, 43 μmol, 1.2 eq). Yield 36 mg, 36 μmol (quantitative). HRMS‐ESI: *m/z* calcd for C_34_H_62_O_6_N_9_Gd [M+H]^2+^, 425.20282 found 425.20202. IR (cm^−1^): 638.9, 722.6, 776.4, 833.2, 937.9, 1024.6, 1078.4, 1129.3, 1171.1, 1224.3, 1248.9, 1317.6, 1390.5, 1607.3, 1736.3, 1766.2, 2863.6, 2977.2, 3281.4.

**[Eu(L_3_)](Cl)**. From **[Eu(L_2_)]**(Cl) (22.5 mg, 31 μmol, 1 eq) with sodium ascorbate (7.4 mg, 37 μmol, 1.2 eq), octane‐azide (9 mg, 45 μmol, 1.5 eq) and copper sulfate pentahydrate (0.77 mg, 3.1 μmol, 0.1 eq) with sodium ascorbate (7.4 mg, 37 μmol, 1.2 eq). Yield 25 mg (92%). HRMS‐ESI: *m/z* calcd for C_34_H_62_O_6_N_9_Eu [M+H]^2+^, 422.70138 found 422.70059. IR (cm^−1^, neat): 636.5, 725.6, 772.2, 939.6, 1024.1, 1076.9, 1128.5, 1172.0, 1224.7, 1247.8, 1278.5, 1318.4, 1390.3, 1598.6, 1732.4, 1760.1, 2869.0, 2977.6, 3311.1.

## Conflict of interest

The authors declare no conflict of interest.

1

## Supporting information

As a service to our authors and readers, this journal provides supporting information supplied by the authors. Such materials are peer reviewed and may be re‐organized for online delivery, but are not copy‐edited or typeset. Technical support issues arising from supporting information (other than missing files) should be addressed to the authors.

Supporting Information

## Data Availability

The data that support the findings of this study are available in the supplementary material of this article.
