# Supplementary material for: Lanthanide Complexes (GdIII and EuIII) Based on a DOTA‐TEMPO Platform for Redox Monitoring via Relaxivity
Source: Chem Asian J. 2022 Jul 28;17(17):e202200544. doi: 10.1002/asia.202200544 (PMC9544908; doi:10.1002/asia.202200544)
Supplement: Supplementary file 1 — Supporting Information [file ASIA-17-0-s001.pdf]

CHEMISTRY
AN ASIAN JOURNAL

Supporting Information

Lanthanide Complexes (Gd^{III} and Eu^{III}) Based on a DOTA-TEMPO Platform for Redox Monitoring via Relaxivity

Richard Barré, Damien Mouchel dit Leguerrier, Quentin Ruet, Lionel Fedele, Daniel Imbert, Véronique Martel-Frachet, Pascal H. Fries, Jennifer K. Molloy,* and Fabrice Thomas*This manuscript is part of a special collection on Responsive Probes and Molecular Bioimaging.© 2022 The Authors. Chemistry – An Asian Journal published by Wiley-VCH GmbH. This is an open access article under the terms of the Creative Commons Attribution License, which permits use, distribution and reproduction in any medium, provided the original work is properly cited.

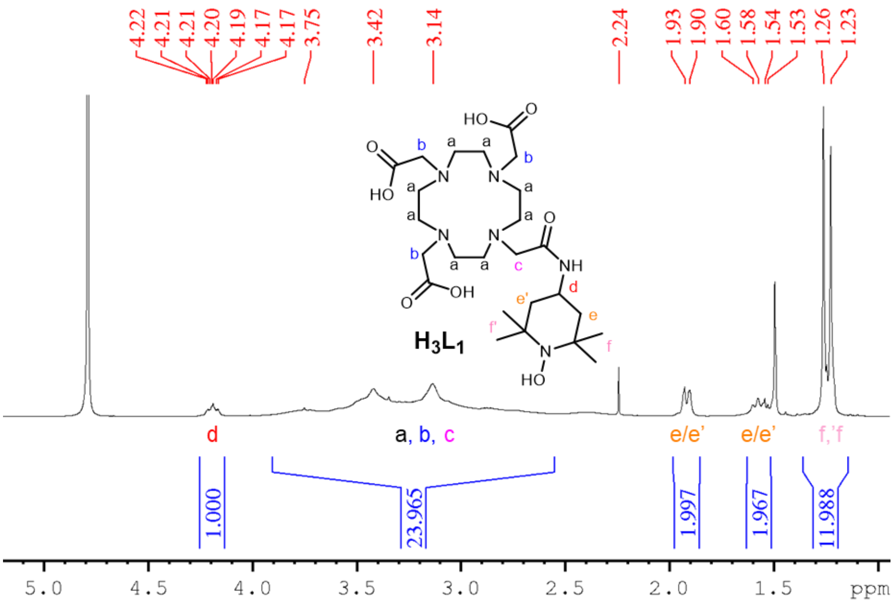

Fig. S1 ^{1}H NMR spectrum of $\mathbf{L_1}$ (400 MHz, D_2O)

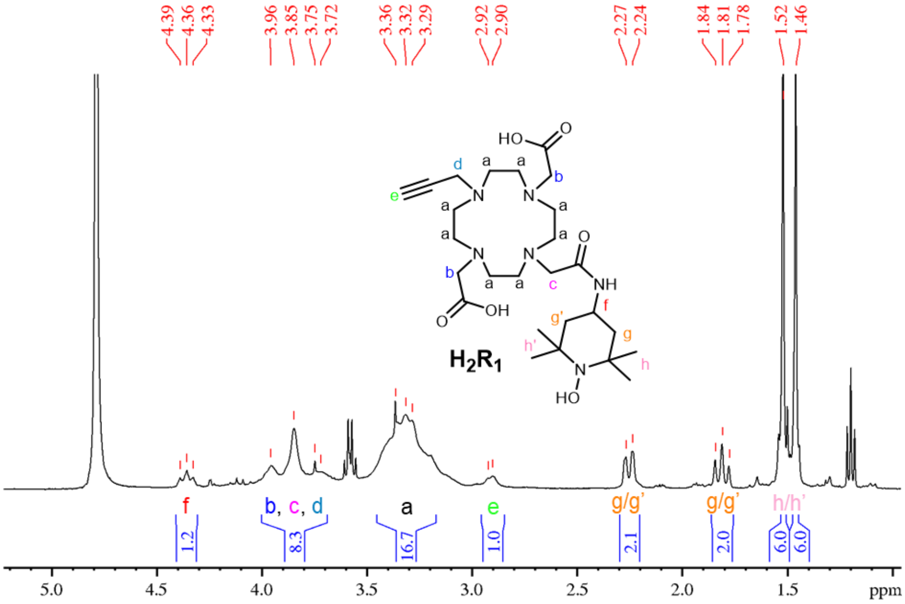

Fig. S2 ^{1}H NMR spectrum of $\mathbf{L_2}$ (400 MHz, D_2O)

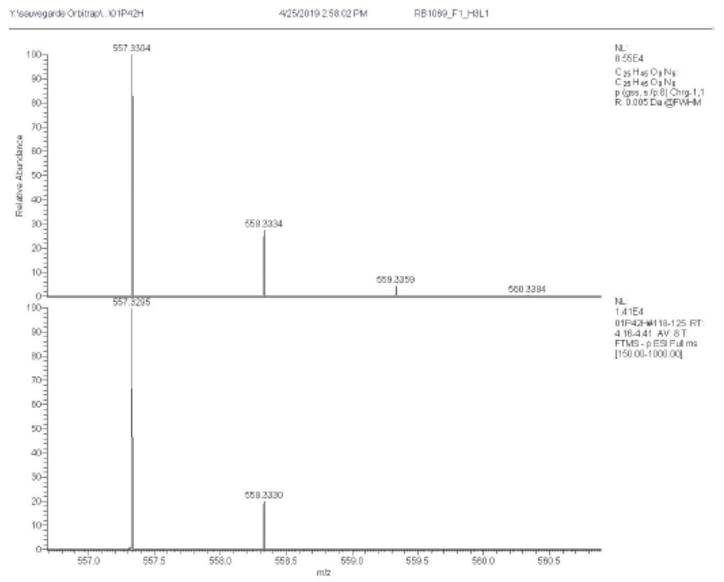

Fig. S3 HR-MS profiles of $\mathbf{L_1}$. Bottom, experimental spectra; top, calculated spectra.

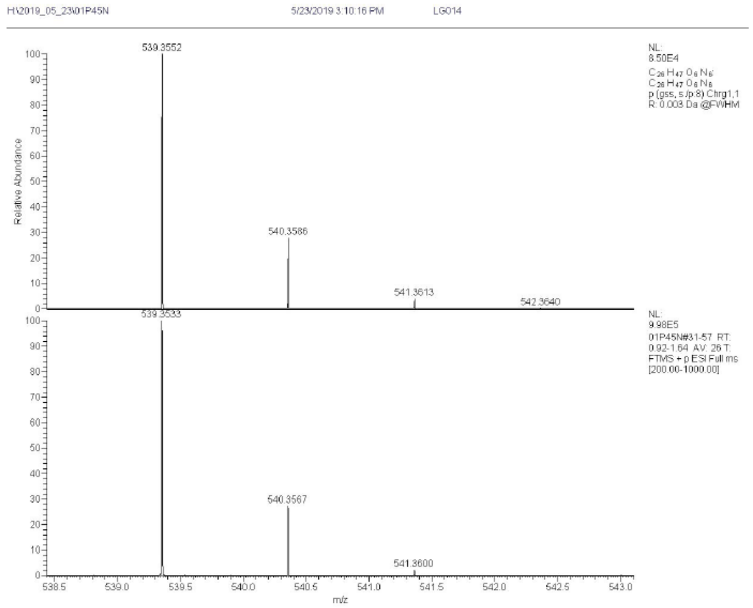

Fig. S4 HR-MS profiles of $\mathbf{L_2}$. Bottom, experimental spectra; top, calculated spectra.

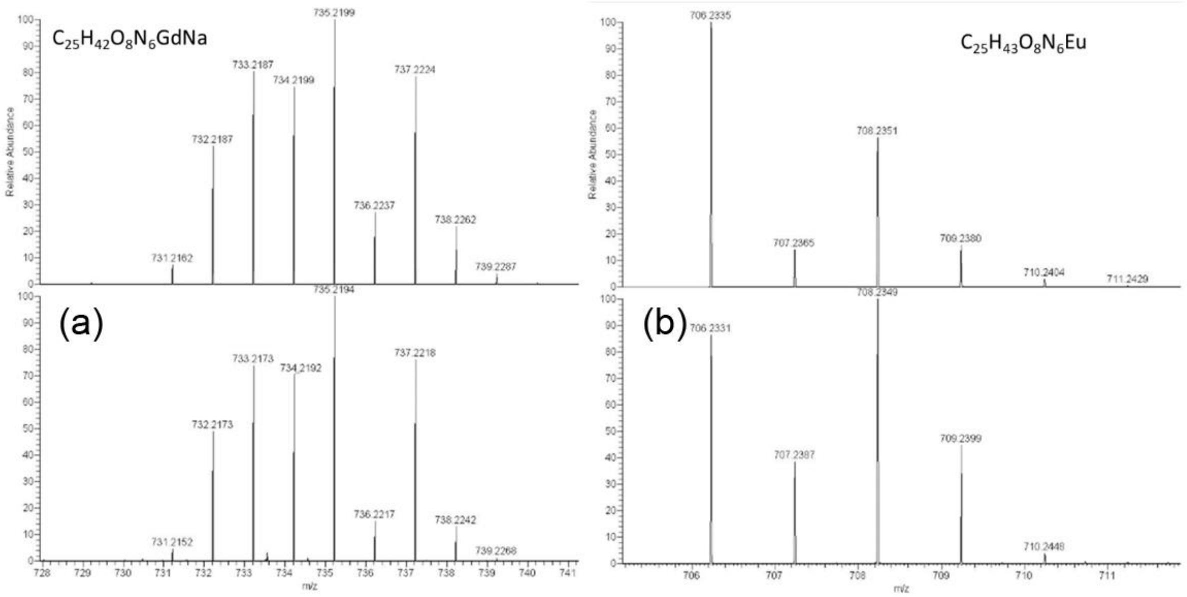

Fig. S5 HR-MS profiles of (a) **[Gd(L$_1$)]** and (b) **[Eu(L$_1$)]**. Bottom, experimental spectra; top, calculated spectra.

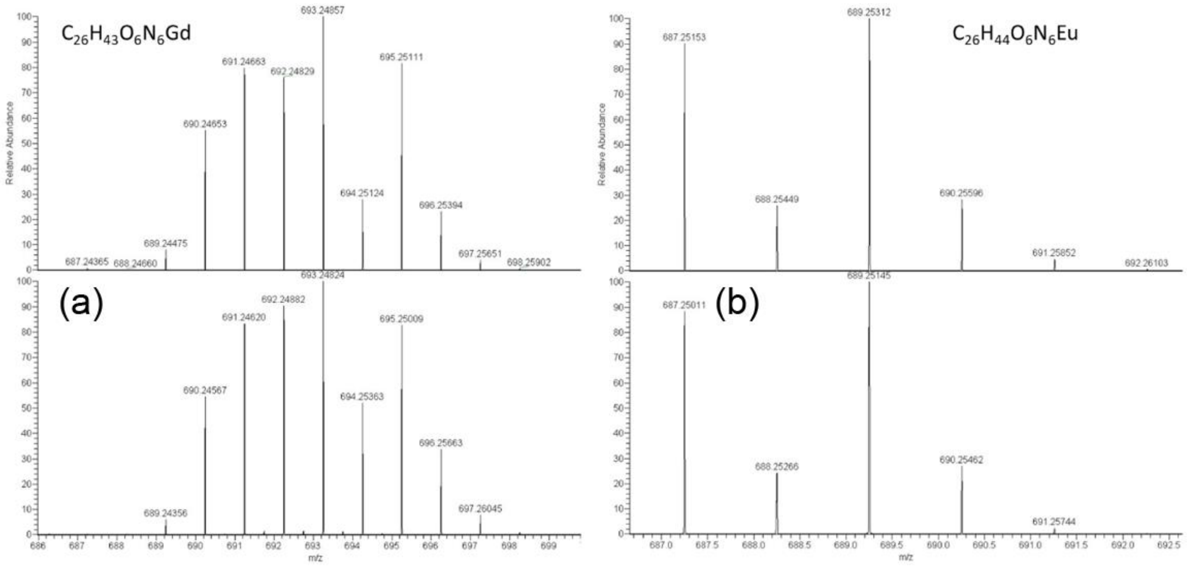

Fig. S6 HR-MS profiles of (a) **[Gd(L$_2$)]$^+$** and (b) **[Eu(L$_2$)]$^+$**. Bottom, experimental spectra; top, calculated spectra.

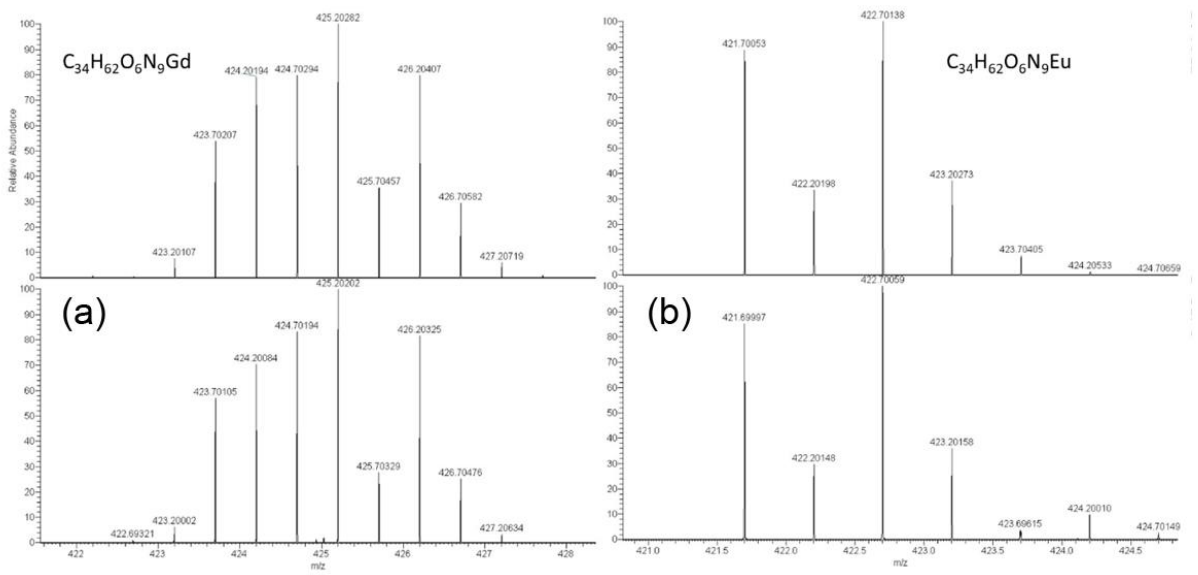

Fig. S7 HR-MS profiles of (a) **[Gd(L$_3$)]**$^+$ and (b) **[Eu(L$_3$)]**$^+$. Bottom, experimental spectra; top, calculated spectra.

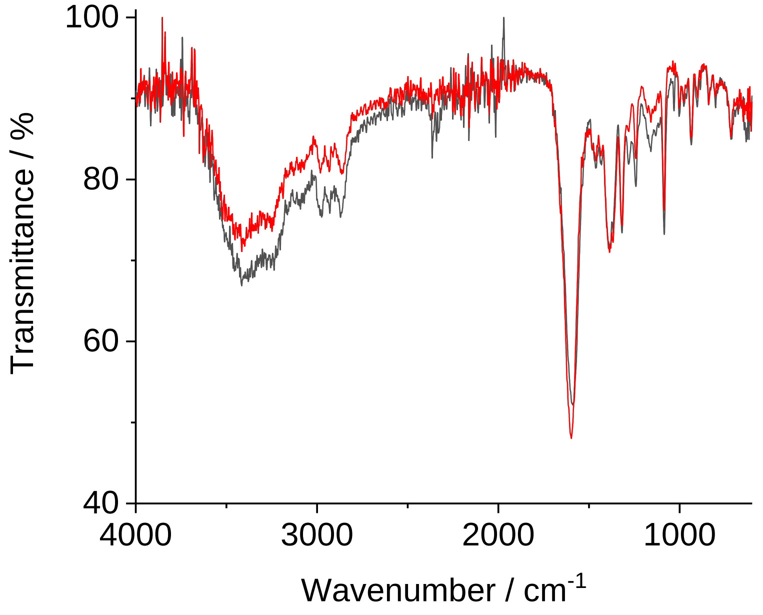

Fig. S8 FTIR spectra of : (black) **[Gd(L$_1$)]** ; (red), **[Eu(L$_1$)]**.

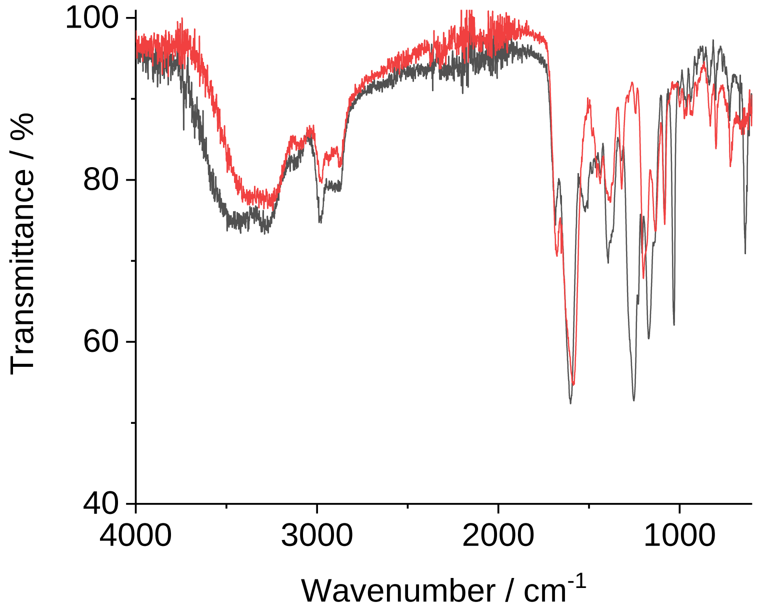

Fig. S9 FTIR spectra of : (black), **[Gd(L$_2$)]$^+$** ; (red), **[Eu(L$_2$)]$^+$**.

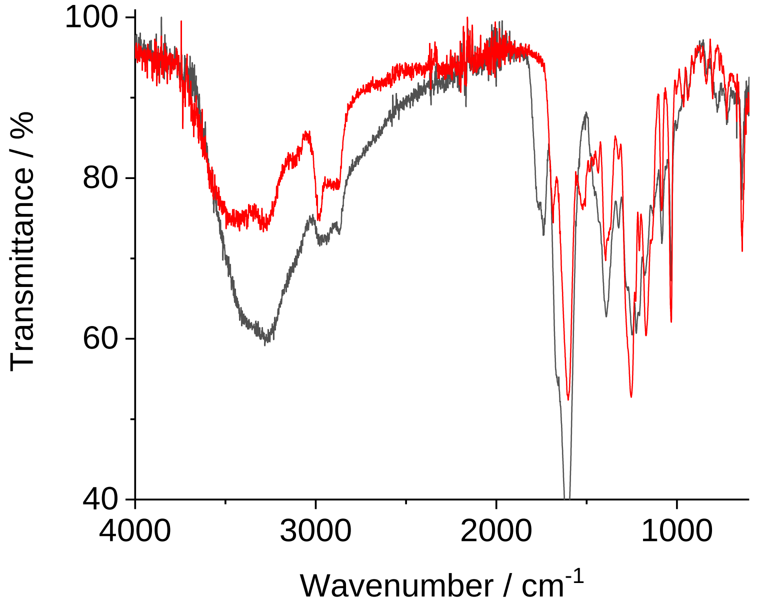

Fig. S10 FTIR spectra of : (black), **[Gd(L$_3$)]$^+$** ; (red), **[Eu(L$_3$)]$^+$**.

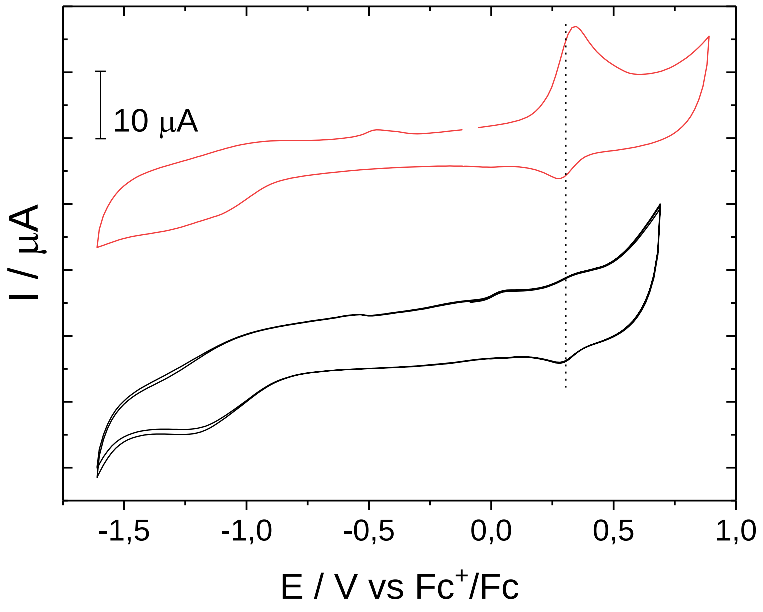

Fig. S11 Cyclic voltammetry curves of (red) **L_1** and (black) **L_2** recorded in CH_3CN (+0.1 M TBAP) at a carbon electrode. Scan rate 0.1 V/s, ref. Fc^+/Fc. Note that **L_2** is less soluble than **L_1** in CH_3CN, resulting in a wave of lower intensity.

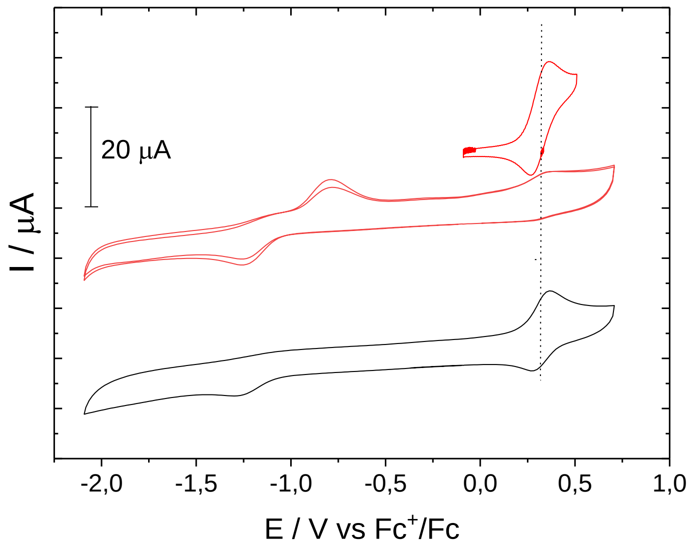

Fig. S12 Cyclic voltammetry curve of (red) **$[Eu(L_2)]^+$** and (black) **$[Gd(L_2)]^+$** recorded in CH_3CN (+0.1 M TBAP) at a carbon electrode. Scan rate 0.1 V/s, ref. Fc^+/Fc.

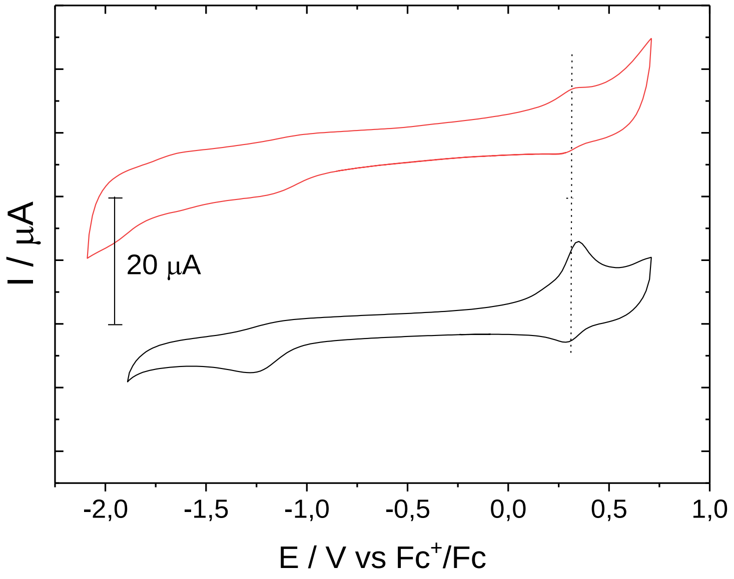

Fig. S13 Cyclic voltammetry curve of (red) **[Eu(L$_3$)]$^+$** and (black) **[Gd(L$_3$)]$^+$** recorded in CH_3CN (+0.1 M TBAP) at a carbon electrode. Scan rate 0.1 V/s, ref. Fc^+/Fc.

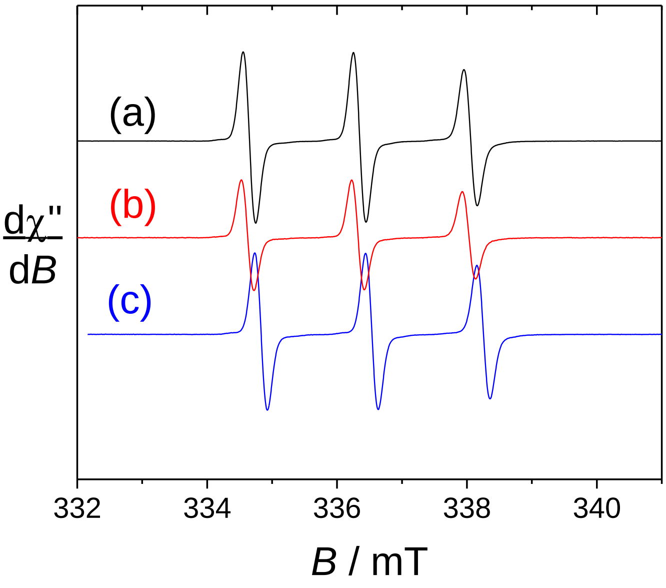

Fig. S14 Isotropic EPR spectra of 0.5 mM solutions of (a) **[Eu(L$_1$)$^•$]** , (b) **[Eu(L$_2$)$^•$]$^+$** and (c) **[Eu(L$_3$)$^•$]$^+$** in 0.1 M HEPES buffer (pH = 7). Microwave Freq. 9.44 GHz ; power, 3.4 mW ; Mod. Amp. 0.2 mT ; Freq. 100 KHz. *T* = 293 K.

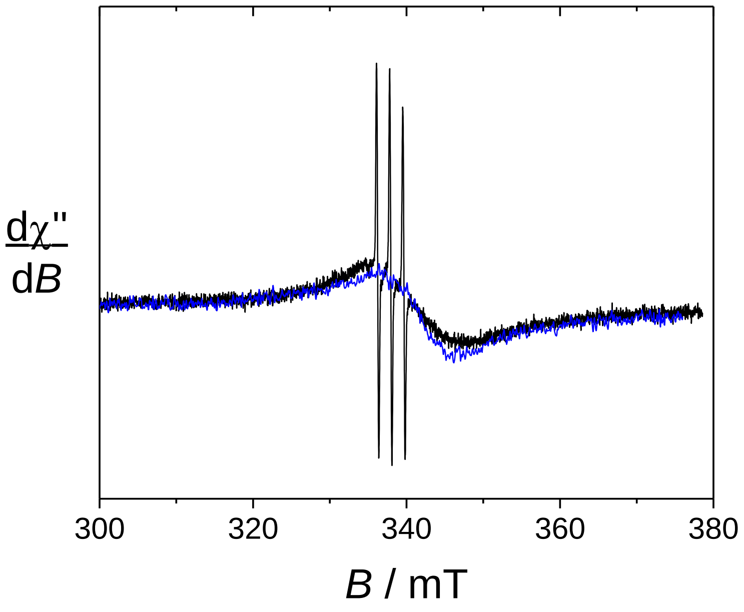

Fig. S15 X-Band EPR spectrum of an aqueous 0.5 mM solution of (black) **[Gd(L$_1$)$^•$]** and (blue) **[Gd(L$_1$)]** (generated from **[Gd(L$_1$)$^•$]** by adding two molar equivalents of sodium ascorbate). Note that this complex is the only one that shows clearly the lanthanide-based resonances at room temperature. Microwave Freq. 9.44 GHz ; power, 3.4 mW ; Mod. Amp. 0.2 mT ; Freq. 100 KHz. *T* = 293 K.

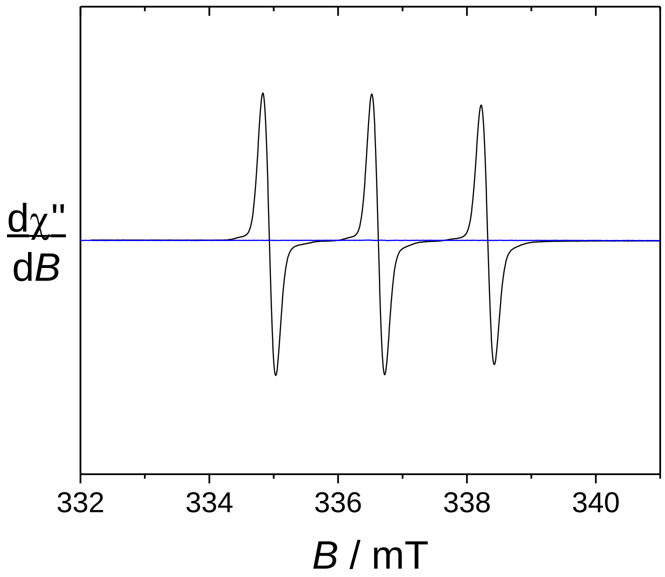

Fig. S16 X-Band EPR spectrum of an aqueous 2 mM solution of (black) **[Gd(L$_2$)$^•$]$^+$** and (blue) **[Gd(L$_2$)]$^+$** (generated from **[Gd(L$_2$)$^•$]$^+$** by adding two molar equivalents of sodium ascorbate). Microwave Freq. 9.44 GHz ; power, 3.4 mW ; Mod. Amp. 0.2 mT ; Freq. 100 KHz. *T* = 293 K.

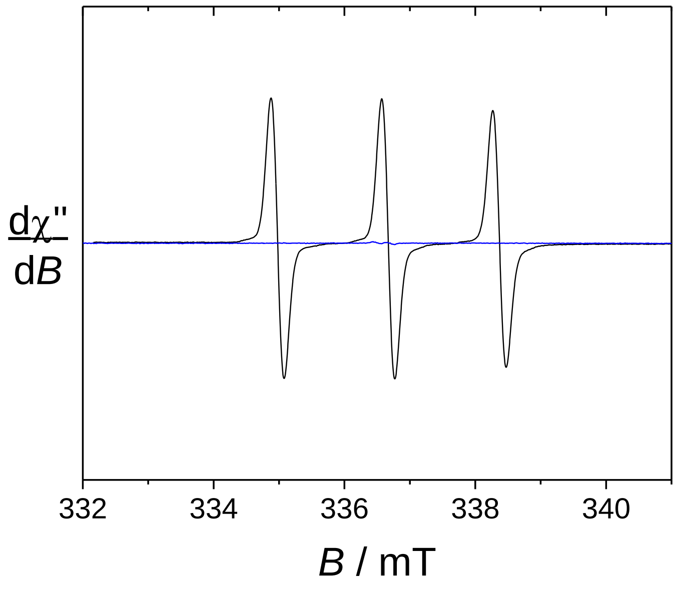

Fig. S17 X-Band EPR spectrum of an aqueous 2 mM solution of (black) **[Gd(L$_3$)$^•$]$^+$** and (blue) **[Gd(L$_3$)]$^+$** (generated from **[Gd(L$_3$)$^•$]$^+$** by adding two molar equivalents of sodium ascorbate). Microwave Freq. 9.44 GHz ; power, 3.4 mW ; Mod. Amp. 0.2 mT ; Freq. 100 KHz. *T* = 293 K.

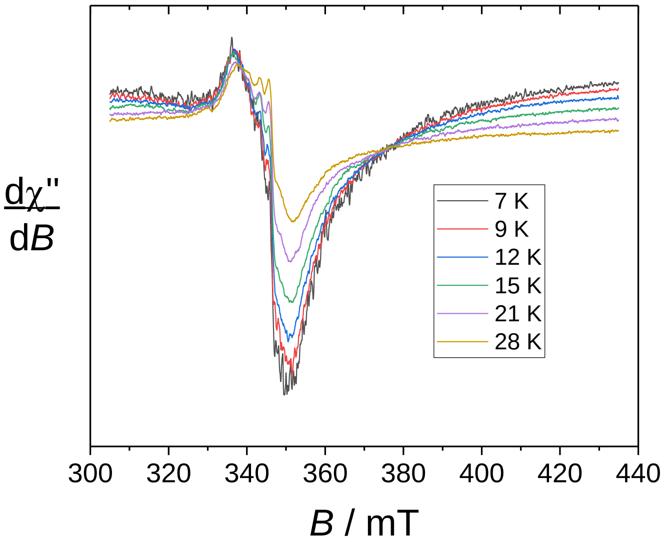

Fig. S18 X-band EPR spectrum of a 0.45 mM aqueous solution (containing 10% glycerol) of **[Gd(L$_1$)$^•$]$^{3+}$**. Microwave Freq; 9.63 GHz, power 0.8 mW; Mod. Freq. 100 KHz, Amp. 0.4 mT. The significant noise at low temperature is a consequence of the saturation.

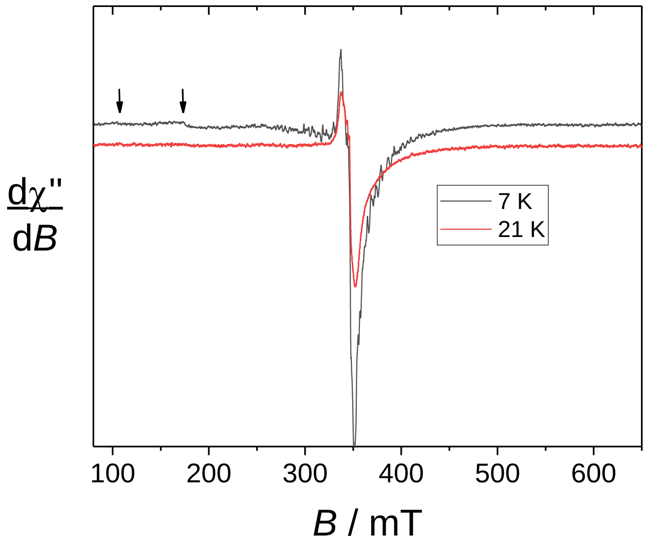

Fig. S19 X-band EPR spectrum of a 0.45 mM aqueous solution (containing 10% glycerol) of **[Gd(L$_1$)$^•$]$^{3+}$**. Microwave Freq; 9.63 GHz, power 0.8 mW; Mod. Freq. 100 KHz, Amp. 0.4 mT.

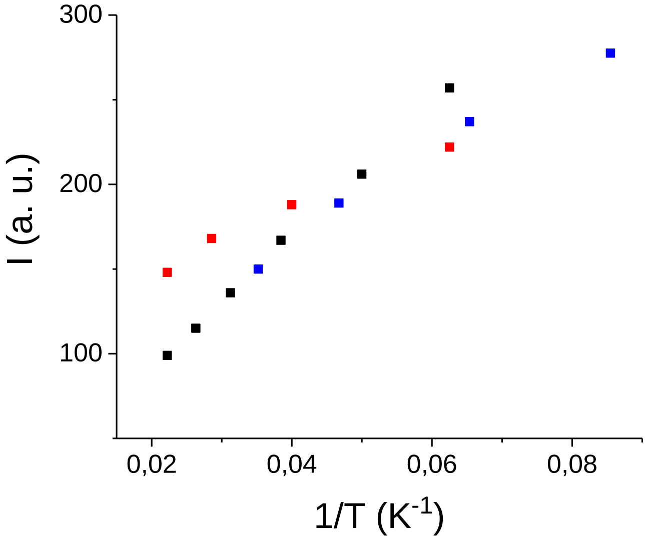

Fig. S20 Intensity of the gadolinium resonance (in the EPR spectrum) as a function of 1/T for complexes: (blue) **[Gd(L$_1$)$^•$]**, (black) **[Gd(L$_2$)$^•$]$^+$** and (red) **[Gd(L$_3$)$^•$]$^+$**. The intensities in the saturated regions were not included in the graph.

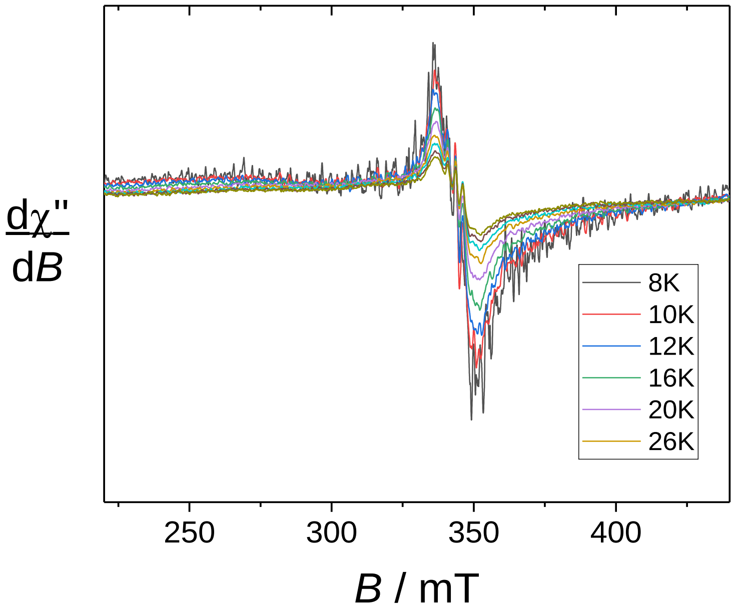

Fig. S21 Low temperature evolution of the EPR spectrum of a 0.45 mM aqueous solution (containing 10% glycerol) of **[Gd(L$_2$)$^•$]$^+$**. Microwave Freq; 9.63 GHz, power 4 mW; Mod. Freq. 100 KHz, Amp. 0.4 mT. The significant noise at low temperature is a consequence of the saturation.

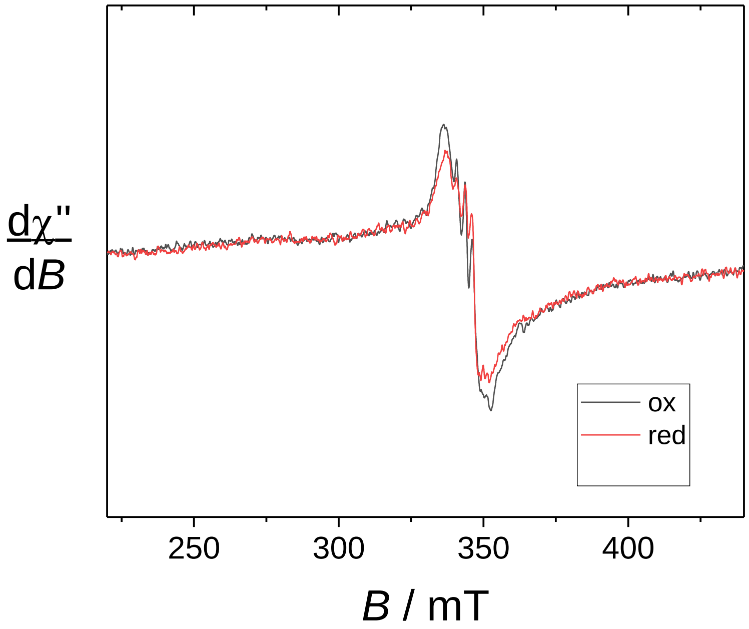

Fig. S22 Low temperature evolution of the EPR spectrum of a 0.45 mM aqueous solution (containing 10% glycerol) of **[Gd(L$_2$)$^•$]$^+$** : (black) before and (red) after addition of two molar equivalents of sodium ascorbate. Microwave Freq; 9.63 GHz, power 4 mW; Mod. Freq. 100 KHz, Amp. 0.4 mT.

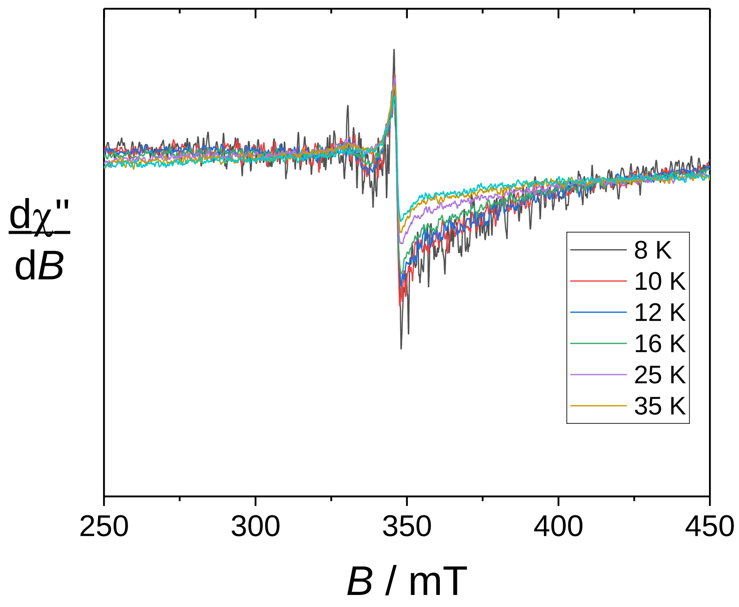

Fig. S23 Low temperature evolution of the EPR spectrum of a 0.45 mM aqueous solution (containing 10% glycerol) of **[Gd(L$_3$)$^•$]$^+$**. Microwave Freq; 9.63 GHz, power 4 mW; Mod. Freq. 100 KHz, Amp. 0.4 mT.

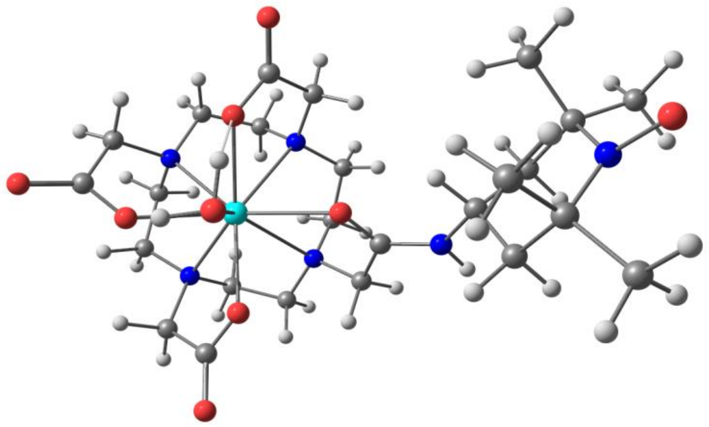

Fig. S24 DFT optimized structure of **[Y(L$_1$)$^•$]**

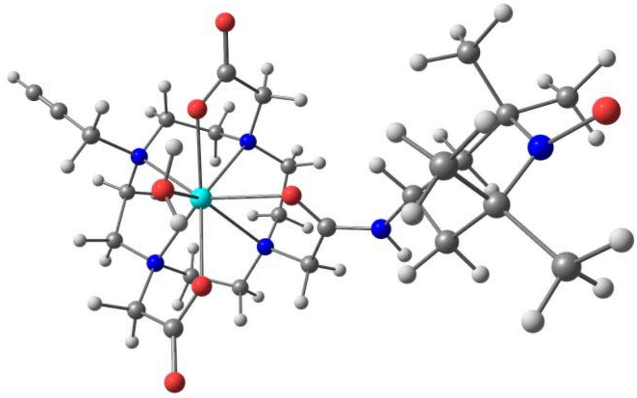

Fig. S25 DFT optimized structure of $\mathbf{[Y(L_2)^{\bullet}]^+}$

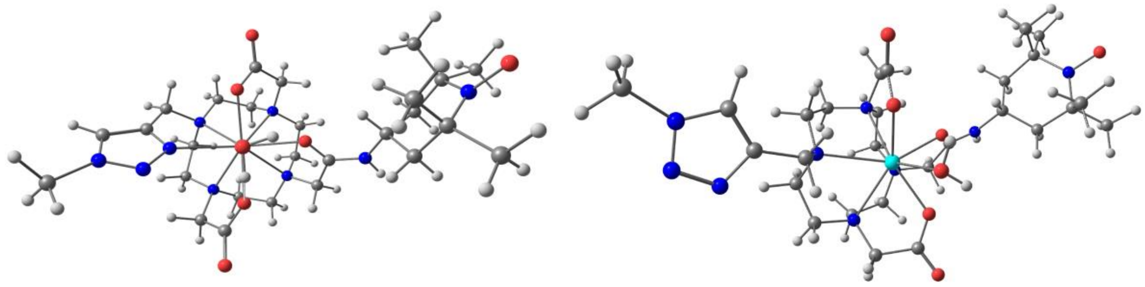

Fig. S26 DFT optimized structures of $\mathbf{[Y(L_3)^{\bullet}]^+}$ with (left) bound or (right) unbound triazole.

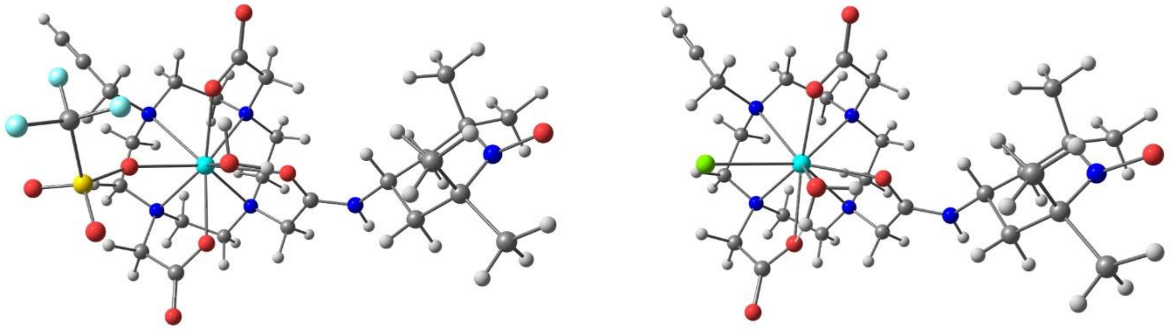

Fig. S27 DFT optimized structures of the triflate (left) and chloride (right) adducts of $\mathbf{[Y(L_2)^{\bullet}]^+}$.

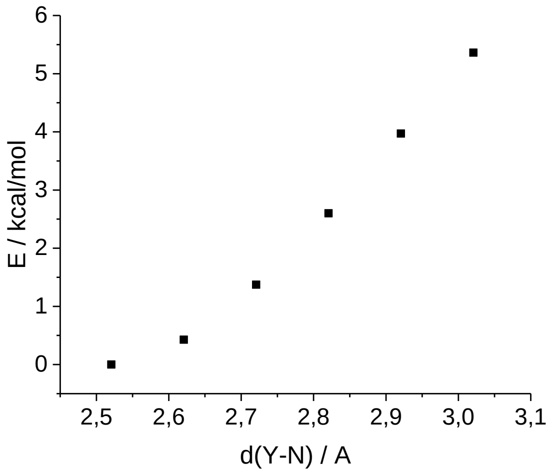

Fig. S28 Energy change upon elongation of the Y-N(triazole) bond. From a relaxed PES of **$[Y(L_3)^{\bullet}]^{+}$**.

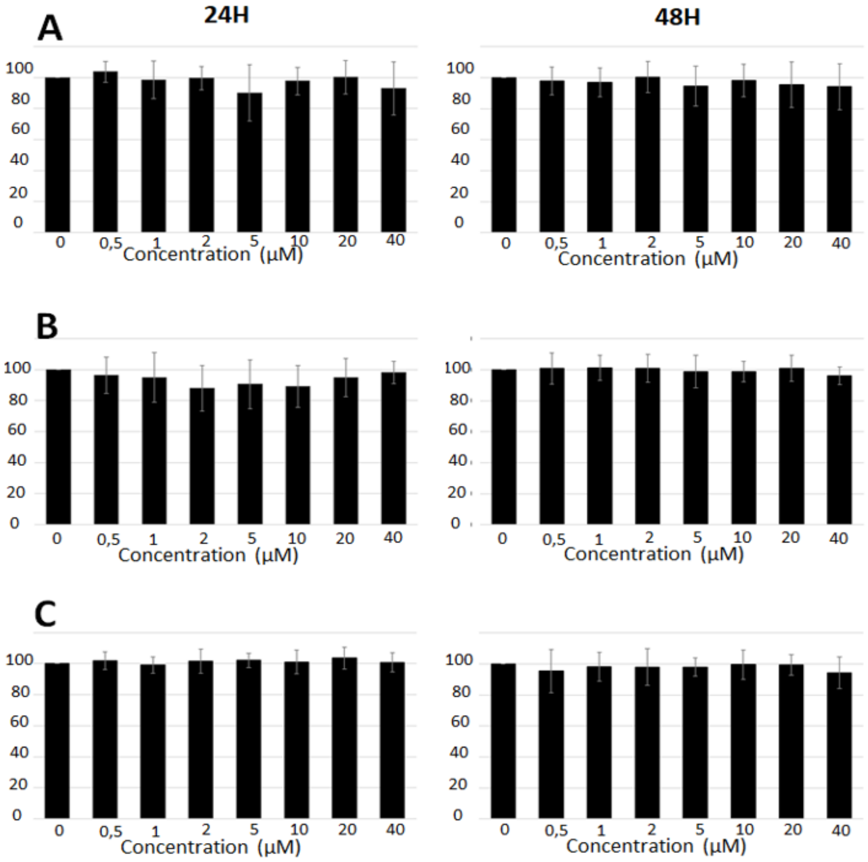

Fig. S29 Cellular viability of M21 cells after 24h and 48h treatment with increasing amount of gadolinium complexes (from MTT assays). (A) **$[Gd(L_2)]$**, (B) **$[Gd(L_2)]^{+}$** and (C) **$[Gd(L_3)]^{+}$**

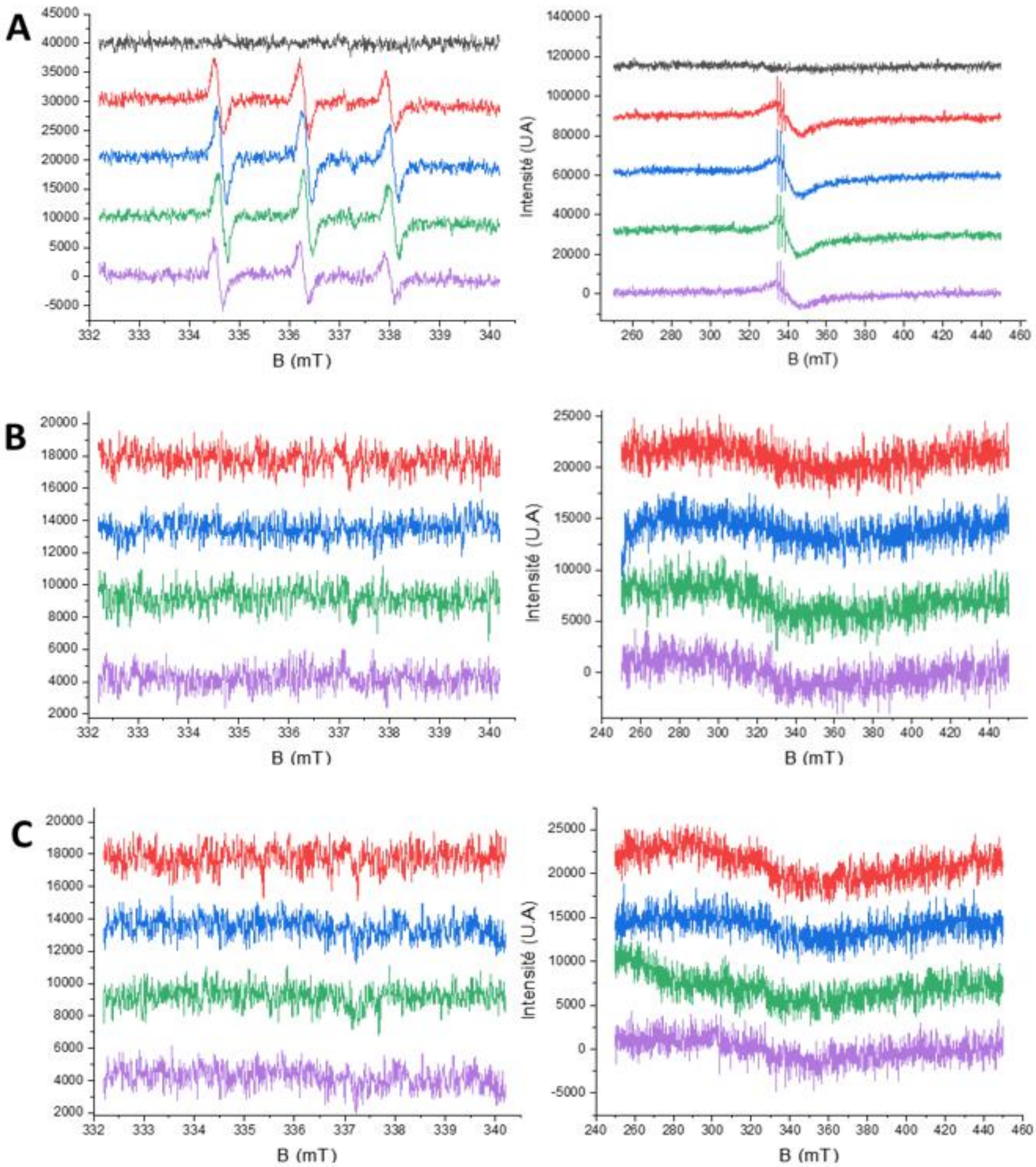

Fig. S30 Isotropic EPR spectra of 0.2 mM solutions of **[Gd(L$_1$)$^{\bullet}$]** incubated with M21 cells at differents time : (black) initial, (red) 5 min, (blue) 10 min, (green) 30 min, (purple) 60 min. (A) supernatant (B) cell pellets, (C) lysates of complex. (left) zoom on the nitroxide resonances, (right) full scale spectra showing the gadolinium lines. Microwave Freq. 9.42 GHz ; power, 2.7 mW ; Mod. Amp. 0.15 mT ; Freq. 100 KHz. *T* = 293 K.

Table S1. Energy difference between eight-coordinate **$[Y(L_2)^{\bullet}]^+$** and nine-coordinate **$[Y(L_2)^{\bullet}]^+$** (including a bound counter-ion).

	Chloride	triflate
free counter-ion (in Ha)	-460,384444	-961,591343
**$[Y(L_2)^{\bullet}]^+$** without counter ion (in Ha, Fig. S25)	-1910,264964	-1910,264964
Sum of energy of free counter-ion and complex **$[Y(L_2)^{\bullet}]^+$** (Ha)	-2370,649408	-2871,856307
**$[Y(L_2)^{\bullet}]^+$** optimized with bound counter-ion (in Ha, Fig. S26)	-2370,641652	-2871,843041
Most stable form	Unbound counter-ion (Fig. S25)	Unbound counter-ion (Fig. S25)
Energy difference between bound and unbound counter ion (in Ha)	0,007756	0,013266
Energy difference between bound and unbound counter ion (in kcal/mol)	4,9	8,4

[1] See Fig S25 and S26 for the optimized structures.

[2] Sum of electronic and thermal free energies from a UB3LYP/6-31g*(LanL2DZ ECP for Y)/PCM calculation.

XYZ coordinates (B3LYP/6-31g*-LANL2DZ for Y/PCM)

Lowest energy conformation of [Y(L$_1$)$^•$] (from a relaxed PES)

Y	-1.606960000	0.058468000	-0.437022000
O	0.782605000	0.305537000	0.077640000
O	-0.871468000	-1.864807000	-1.418359000
O	7.403475000	-0.306145000	-0.949515000
O	-1.474073000	2.375843000	-0.684459000
N	6.296587000	0.051689000	-0.401537000
N	-1.049683000	1.082007000	2.039679000
N	-3.706240000	1.339473000	0.609459000
N	2.524666000	1.108500000	1.299372000
H	2.793081000	1.669002000	2.100713000
N	-0.993533000	-1.859179000	1.360656000
N	-3.648714000	-1.602490000	-0.078627000
O	-2.403737000	4.420680000	-0.819623000
O	0.040230000	-3.922250000	-1.437744000
C	0.260101000	1.750763000	1.928751000
H	0.703596000	1.954432000	2.913996000
H	0.111940000	2.710710000	1.425005000
C	3.602913000	0.534260000	0.485832000
H	3.108773000	-0.131594000	-0.223597000
C	1.222176000	0.980847000	1.037946000
C	-0.325658000	-2.895261000	-0.859655000
C	-3.807353000	2.605720000	-0.155429000
H	-4.395575000	3.358049000	0.387140000
H	-4.329484000	2.392615000	-1.092752000
C	-2.457690000	3.215302000	-0.571357000
C	5.507740000	1.094341000	-1.142856000
C	4.571111000	-0.247174000	1.377673000
H	4.999142000	0.443484000	2.116676000
H	4.034264000	-1.022762000	1.934479000
C	4.371820000	1.636597000	-0.250746000
H	3.689948000	2.222008000	-0.877015000
H	4.794504000	2.322387000	0.495196000
C	5.716335000	-0.916425000	0.590308000
C	-2.093174000	2.080924000	2.402894000
H	-1.869187000	3.012541000	1.881992000
H	-2.056906000	2.305177000	3.479414000
C	-0.059652000	-2.768138000	0.651414000
H	-0.046975000	-3.766962000	1.107378000
H	0.950195000	-2.355823000	0.738112000
C	-0.351593000	-1.292152000	2.575411000
H	0.700829000	-1.111106000	2.352536000
H	-0.369446000	-2.016186000	3.404224000
C	4.959479000	0.484532000	-2.451182000
H	5.775815000	0.013182000	-3.005979000
H	4.524855000	1.274113000	-3.073516000
H	4.185127000	-0.267207000	-2.272325000
C	6.470909000	2.244715000	-1.481724000
H	6.928154000	2.648139000	-0.571979000

H	5.916866000	3.049192000	-1.976488000
H	7.264877000	1.902384000	-2.148084000
C	-4.145740000	-1.878308000	-1.447863000
H	-5.177834000	-2.253230000	-1.433982000
H	-3.516293000	-2.658606000	-1.886060000
C	6.838854000	-1.300811000	1.568782000
H	6.435718000	-1.960343000	2.344299000
H	7.252966000	-0.409983000	2.052839000
H	7.645597000	-1.820863000	1.048740000
C	-4.931547000	0.515948000	0.424273000
H	-5.260756000	0.625111000	-0.609628000
H	-5.751799000	0.887452000	1.057312000
C	-4.706483000	-0.959680000	0.744964000
H	-4.434081000	-1.070867000	1.796484000
H	-5.660832000	-1.493419000	0.615903000
C	5.229826000	-2.179733000	-0.152079000
H	4.961763000	-2.954803000	0.573842000
H	6.032314000	-2.562249000	-0.789195000
H	4.353354000	-1.988752000	-0.778421000
C	-3.184419000	-2.851449000	0.580788000
H	-2.691092000	-3.469122000	-0.171217000
H	-4.037805000	-3.435594000	0.957785000
C	-3.503549000	1.615825000	2.053552000
H	-3.744057000	0.709203000	2.612324000
H	-4.213556000	2.385040000	2.395648000
C	-4.046671000	-0.676249000	-2.405203000
C	-2.232127000	-2.586767000	1.744763000
H	-2.743269000	-2.008087000	2.516861000
H	-1.972625000	-3.551826000	2.207262000
C	-1.017148000	-0.004505000	3.056154000
H	-2.046812000	-0.214349000	3.350696000
H	-0.500351000	0.339329000	3.965205000
O	-3.111455000	0.176487000	-2.135154000
O	-0.206938000	0.868120000	-2.417268000
H	-0.450133000	1.790749000	-2.187337000
H	0.693251000	0.756707000	-2.063946000
O	-4.809759000	-0.626745000	-3.373289000

Lowest energy conformation of $[Y(L_2)^{\bullet}]^+$ (from a relaxed PES)

Y	-1.517406000	0.194167000	-0.469766000
O	0.757129000	0.225391000	0.107241000
O	-1.024727000	-1.893415000	-1.241662000
O	7.365263000	-0.728360000	-0.881084000
O	-1.034284000	2.334959000	-1.076975000
N	6.271875000	-0.288867000	-0.366919000
N	-1.055035000	1.432369000	1.828424000
N	-3.503776000	1.826173000	0.068016000
N	2.549102000	1.067254000	1.222934000
H	2.854207000	1.700379000	1.954957000
N	-1.353461000	-1.542406000	1.485255000
N	-3.842744000	-1.138305000	-0.243457000

O	-1.556718000	4.497964000	-1.415874000
O	-0.370635000	-4.030002000	-0.975341000
C	0.338080000	1.920319000	1.771823000
H	0.744888000	2.114190000	2.773316000
H	0.352436000	2.865976000	1.222266000
C	3.595024000	0.386343000	0.448125000
H	3.065894000	-0.258264000	-0.255191000
C	1.243026000	0.986784000	0.981754000
C	-0.656846000	-2.918868000	-0.532921000
C	-3.326456000	2.998094000	-0.829822000
H	-3.843335000	3.883722000	-0.439254000
H	-3.778270000	2.757925000	-1.798325000
C	-1.853505000	3.340993000	-1.120532000
C	5.568851000	0.784987000	-1.147766000
C	4.489312000	-0.439684000	1.376561000
H	4.943836000	0.235754000	2.113159000
H	3.890822000	-1.168591000	1.933585000
C	4.451676000	1.418221000	-0.293622000
H	3.823753000	2.034271000	-0.946320000
H	4.902972000	2.089832000	0.448472000
C	5.606992000	-1.196655000	0.629204000
C	-1.997784000	2.583673000	1.956490000
H	-1.605004000	3.415048000	1.370305000
H	-2.045169000	2.927440000	2.999207000
C	-0.514280000	-2.658915000	0.976597000
H	-0.704608000	-3.587241000	1.529695000
H	0.536392000	-2.395881000	1.133080000
C	-0.729878000	-0.923036000	2.687379000
H	0.350131000	-0.899658000	2.532880000
H	-0.904213000	-1.540113000	3.580593000
C	5.010023000	0.184373000	-2.455714000
H	5.804226000	-0.354515000	-2.980099000
H	4.645624000	0.987709000	-3.105067000
H	4.180798000	-0.507538000	-2.279990000
C	6.610699000	1.864938000	-1.485877000
H	7.072681000	2.256669000	-0.573379000
H	6.120671000	2.693068000	-2.008387000
H	7.395896000	1.459784000	-2.126984000
C	-4.238187000	-1.572046000	-1.619514000
C	6.680015000	-1.631462000	1.641396000
H	6.219142000	-2.250733000	2.417951000
H	7.137481000	-0.759179000	2.120020000
H	7.464688000	-2.209774000	1.149813000
C	-4.820209000	1.171555000	-0.177947000
H	-4.991367000	1.170816000	-1.257159000
H	-5.637228000	1.754773000	0.271160000
C	-4.882984000	-0.257362000	0.355206000
H	-4.753044000	-0.266378000	1.438730000
H	-5.884786000	-0.664942000	0.162572000
C	5.059432000	-2.441501000	-0.101804000
H	4.726670000	-3.184646000	0.630521000
H	5.851023000	-2.884690000	-0.712673000

H	4.212033000	-2.208232000	-0.752881000
C	-3.596291000	-2.334221000	0.609922000
H	-3.129923000	-3.099683000	-0.013226000
H	-4.546332000	-2.755765000	0.965166000
C	-3.411708000	2.242569000	1.495412000
H	-3.816590000	1.437601000	2.110914000
H	-4.052275000	3.119601000	1.670657000
C	-2.720400000	-2.031085000	1.822176000
H	-3.206601000	-1.279549000	2.447149000
H	-2.648762000	-2.943000000	2.433302000
C	-1.251439000	0.484811000	2.963352000
H	-2.319498000	0.447764000	3.183388000
H	-0.762302000	0.872083000	3.868649000
O	-0.739998000	0.217181000	-2.772672000
H	-0.125951000	0.939169000	-2.988178000
H	-0.291807000	-0.630531000	-2.943632000
C	-5.457764000	-2.388868000	-1.681554000
C	-6.461654000	-3.059580000	-1.735552000
H	-3.390101000	-2.122580000	-2.038162000
H	-4.366429000	-0.678367000	-2.239519000
H	-7.346297000	-3.655755000	-1.787694000

$[Y(L_3)^{\bullet}]^+$ with the triazole moiety bound :

Y	-1.299380000	-0.029758000	-0.026241000
O	1.063125000	-0.080186000	0.365439000
O	-0.726634000	-0.462981000	-2.172257000
O	7.506023000	1.052014000	-1.138074000
O	-1.260215000	1.686984000	1.514586000
N	6.468746000	0.742029000	-0.444443000
N	-0.583141000	-1.197510000	2.305782000
N	-3.365745000	-0.105298000	1.691953000
N	2.905123000	-0.274924000	1.680678000
H	3.249629000	-0.481392000	2.612090000
N	-0.580417000	-2.569731000	-0.369624000
N	-3.364492000	-1.493776000	-1.003689000
O	-2.165944000	3.037722000	3.069631000
O	0.135976000	-1.730256000	-3.819735000
C	0.706491000	-0.588491000	2.689977000
H	1.235940000	-1.189030000	3.442666000
H	0.506656000	0.393334000	3.129862000
C	3.894174000	0.099632000	0.661262000
H	3.328832000	0.203197000	-0.265923000
C	1.588467000	-0.312543000	1.482477000
C	-0.143328000	-1.523480000	-2.638060000
C	-3.529388000	1.281005000	2.194651000
H	-4.048302000	1.299559000	3.161764000
H	-4.149115000	1.835393000	1.483801000
C	-2.216974000	2.076607000	2.303757000
C	5.598603000	1.891488000	-0.021180000
C	4.957687000	-0.993129000	0.527237000
H	5.464772000	-1.107046000	1.494699000

H	4.489028000	-1.954850000	0.292286000
C	4.573278000	1.422119000	1.031541000
H	3.825044000	2.211552000	1.162042000
H	5.079569000	1.293831000	1.997147000
C	6.007815000	-0.683708000	-0.559896000
C	-1.612297000	-0.894213000	3.341447000
H	-1.440415000	0.118640000	3.707318000
H	-1.498296000	-1.564383000	4.205764000
C	0.278508000	-2.560542000	-1.580929000
H	0.333605000	-3.555331000	-2.041340000
H	1.292079000	-2.280424000	-1.278271000
C	0.172556000	-3.086045000	0.805200000
H	1.198660000	-2.721388000	0.738131000
H	0.233238000	-4.184136000	0.779873000
C	4.902403000	2.489665000	-1.263144000
H	5.648778000	2.702912000	-2.033646000
H	4.403272000	3.426276000	-0.992731000
H	4.150024000	1.818930000	-1.688769000
C	6.514240000	2.953457000	0.610377000
H	7.078028000	2.529738000	1.448104000
H	5.905386000	3.781967000	0.986712000
H	7.222048000	3.342483000	-0.124065000
C	-3.976550000	-0.743969000	-2.136735000
C	7.236238000	-1.582769000	-0.339991000
H	6.928396000	-2.633199000	-0.365253000
H	7.696050000	-1.380077000	0.633078000
H	7.982708000	-1.416101000	-1.118872000
C	-4.606149000	-0.577142000	1.015942000
H	-5.032079000	0.260327000	0.463568000
H	-5.360574000	-0.882312000	1.756251000
C	-4.360648000	-1.755889000	0.074054000
H	-4.008090000	-2.613521000	0.649775000
H	-5.322965000	-2.056453000	-0.366393000
C	5.445879000	-0.925184000	-1.977572000
H	5.257940000	-1.994181000	-2.124123000
H	6.176113000	-0.594545000	-2.721642000
H	4.507564000	-0.391615000	-2.156126000
C	-2.828174000	-2.787798000	-1.515115000
H	-2.392334000	-2.610032000	-2.499023000
H	-3.642647000	-3.512969000	-1.658994000
C	-3.039085000	-1.023455000	2.817782000
H	-3.221857000	-2.047355000	2.487484000
H	-3.728254000	-0.844554000	3.657155000
C	-1.784722000	-3.416396000	-0.597033000
H	-2.230079000	-3.644912000	0.372827000
H	-1.485103000	-4.382098000	-1.032060000
C	-0.451346000	-2.671052000	2.135531000
H	-1.446984000	-3.108474000	2.227530000
H	0.147981000	-3.095936000	2.954219000
H	-4.969777000	-1.142707000	-2.384976000
H	-3.337155000	-0.893837000	-3.010910000
C	-4.927411000	1.699605000	-2.312342000

C	-4.055419000	0.727081000	-1.870764000
N	-4.442528000	2.855551000	-1.791526000
N	-3.344490000	2.639705000	-1.064986000
N	-3.106663000	1.347631000	-1.117239000
C	-4.973395000	4.209248000	-1.936042000
H	-4.974618000	4.492150000	-2.990499000
H	-5.989445000	4.249340000	-1.538782000
H	-4.324612000	4.877211000	-1.371064000
H	-5.813369000	1.663199000	-2.926762000
O	-0.181011000	2.074558000	-0.839173000
H	-0.318802000	2.521278000	0.022641000
H	0.765324000	1.848934000	-0.859457000

$[Y(L_3)^{\bullet}]^+$ with the triazole moiety unbound :

Y	-0.866654000	0.583312000	-0.328336000
O	1.410284000	0.319229000	0.155735000
O	-0.742690000	-1.663269000	-0.697268000
O	7.625420000	-1.937705000	-1.051973000
O	-0.055058000	2.462371000	-1.343086000
N	6.677540000	-1.223978000	-0.556739000
N	-0.055556000	2.123778000	1.674366000
N	-2.500228000	2.636561000	-0.053593000
N	3.396772000	1.030314000	0.992036000
H	3.859891000	1.718028000	1.576478000
N	-0.858930000	-0.763122000	1.913932000
N	-3.342601000	-0.240081000	0.198054000
O	-0.233699000	4.564941000	-2.125815000
O	-0.443070000	-3.794425000	-0.038975000
C	1.397877000	2.327608000	1.493871000
H	1.893265000	2.601048000	2.435134000
H	1.547853000	3.154777000	0.793955000
C	4.247183000	0.055078000	0.296051000
H	3.560227000	-0.600359000	-0.240581000
C	2.077565000	1.130362000	0.844854000
C	-0.510777000	-2.586851000	0.186008000
C	-2.183464000	3.561602000	-1.171287000
H	-2.507642000	4.586217000	-0.948897000
H	-2.737801000	3.230983000	-2.056367000
C	-0.697349000	3.565789000	-1.576635000
C	6.084813000	-0.194730000	-1.477491000
C	5.079655000	-0.741325000	1.303294000
H	5.709330000	-0.039747000	1.866361000
H	4.426987000	-1.242077000	2.026364000
C	5.185262000	0.772561000	-0.680421000
H	4.607673000	1.373349000	-1.391270000
H	5.817793000	1.465781000	-0.109883000
C	5.977436000	-1.806805000	0.639149000
C	-0.766129000	3.436541000	1.603284000
H	-0.267800000	4.053436000	0.855021000
H	-0.681637000	3.970480000	2.560055000
C	-0.233556000	-2.076355000	1.609672000

H	-0.527829000	-2.838964000	2.341601000
H	0.852652000	-1.961454000	1.675400000
C	-0.082056000	-0.046479000	2.963324000
H	0.978393000	-0.234974000	2.786385000
H	-0.307594000	-0.448108000	3.961645000
C	5.298386000	-0.904002000	-2.601536000
H	5.933719000	-1.660012000	-3.071683000
H	5.002821000	-0.174024000	-3.362614000
H	4.391114000	-1.394844000	-2.237071000
C	7.249533000	0.597491000	-2.094436000
H	7.866174000	1.052872000	-1.312414000
H	6.850732000	1.395285000	-2.729553000
H	7.881886000	-0.052880000	-2.701877000
C	-3.892489000	-0.824412000	-1.075463000
C	7.061274000	-2.241281000	1.640097000
H	6.585484000	-2.618328000	2.551267000
H	7.702205000	-1.395101000	1.909140000
H	7.685901000	-3.030211000	1.217072000
C	-3.918372000	2.184538000	-0.120594000
H	-4.164937000	2.021210000	-1.172160000
H	-4.597075000	2.968774000	0.245466000
C	-4.170784000	0.904932000	0.671807000
H	-3.952376000	1.065983000	1.729079000
H	-5.238564000	0.661337000	0.608634000
C	5.163284000	-3.042717000	0.199036000
H	4.781333000	-3.568326000	1.080693000
H	5.809690000	-3.724717000	-0.360794000
H	4.309174000	-2.782124000	-0.433113000
C	-3.250010000	-1.293197000	1.246750000
H	-2.955192000	-2.225005000	0.760330000
H	-4.232664000	-1.474506000	1.703553000
C	-2.245039000	3.294349000	1.256679000
H	-2.756538000	2.722200000	2.032665000
H	-2.696841000	4.297744000	1.265215000
C	-2.266928000	-0.951160000	2.365676000
H	-2.587925000	-0.038426000	2.871459000
H	-2.311758000	-1.752157000	3.118324000
C	-0.352382000	1.455028000	2.972743000
H	-1.401203000	1.641107000	3.208818000
H	0.234283000	1.916218000	3.780380000
H	-3.171106000	-1.574625000	-1.407227000
H	-3.904906000	-0.030593000	-1.827364000
C	-5.642868000	-2.743453000	-0.777138000
C	-5.263498000	-1.430505000	-0.990497000
N	-6.994614000	-2.721476000	-0.811396000
N	-7.446149000	-1.469594000	-1.035290000
N	-6.402545000	-0.688755000	-1.145997000
C	-7.933529000	-3.825372000	-0.650825000
H	-7.759396000	-4.580760000	-1.420439000
H	-7.818367000	-4.274552000	0.338327000
H	-8.937922000	-3.415778000	-0.755835000
H	-5.080604000	-3.651426000	-0.620473000

O	-0.123276000	0.057931000	-2.591148000
H	0.575292000	0.663457000	-2.892353000
H	0.235984000	-0.846893000	-2.584924000

[Y(L$_2$)$^{•}$]$^{+}$ from geometry optimization (used for thermochemistry calculations) :

Y	-1,517149000	0,193416000	-0,469711000
O	0,756924000	0,223161000	0,108972000
O	-1,023804000	-1,892651000	-1,241553000
O	7,365357000	-0,728299000	-0,881713000
O	-1,033071000	2,334442000	-1,077896000
N	6,271923000	-0,289215000	-0,367305000
N	-1,055085000	1,431343000	1,829231000
N	-3,502771000	1,826759000	0,068197000
N	2,549214000	1,065990000	1,223423000
H	2,854419000	1,699073000	1,955443000
N	-1,355077000	-1,543399000	1,485506000
N	-3,842812000	-1,137791000	-0,244657000
O	-1,553163000	4,499000000	-1,410556000
O	-0,375358000	-4,031004000	-0,975637000
C	0,338298000	1,918546000	1,773320000
H	0,744942000	2,111610000	2,775044000
H	0,353363000	2,864559000	1,224386000
C	3,595051000	0,385400000	0,448241000
H	3,065857000	-0,258761000	-0,255447000
C	1,243053000	0,984961000	0,983002000
C	-0,658611000	-2,919115000	-0,533050000
C	-3,324528000	2,999126000	-0,828858000
H	-3,841297000	3,884717000	-0,438060000
H	-3,775822000	2,759673000	-1,797786000
C	-1,851282000	3,341567000	-1,118544000
C	5,568872000	0,785119000	-1,147457000
C	4,489382000	-0,441212000	1,376119000
H	4,943895000	0,233780000	2,113133000
H	3,890929000	-1,170491000	1,932693000
C	4,451678000	1,417746000	-0,292898000
H	3,823724000	2,034202000	-0,945184000
H	4,902972000	2,088903000	0,449610000
C	5,607058000	-1,197667000	0,628236000
C	-1,997370000	2,582970000	1,957401000
H	-1,604162000	3,414427000	1,371596000
H	-2,044940000	2,926455000	3,000205000
C	-0,516237000	-2,660062000	0,976703000
H	-0,707233000	-3,588556000	1,529304000
H	0,534495000	-2,397568000	1,133667000
C	-0,731634000	-0,924506000	2,687845000
H	0,348438000	-0,901698000	2,533710000
H	-0,906602000	-1,541528000	3,580985000
C	5,010098000	0,185376000	-2,455821000
H	5,804350000	-0,353089000	-2,980569000
H	4,645651000	0,989137000	-3,104620000
H	4,180908000	-0,506707000	-2,280596000

C	6,610684000	1,865340000	-1,484825000
H	7,072663000	2,256443000	-0,572054000
H	6,120629000	2,693828000	-2,006741000
H	7,395896000	1,460677000	-2,126223000
C	-4,237571000	-1,570963000	-1,621082000
C	6,680107000	-1,633126000	1,640124000
H	6,219247000	-2,252852000	2,416321000
H	7,137628000	-0,761153000	2,119258000
H	7,464730000	-2,211169000	1,148146000
C	-4,819197000	1,172645000	-0,179020000
H	-4,989460000	1,172371000	-1,258375000
H	-5,636413000	1,755970000	0,269612000
C	-4,882983000	-0,256422000	0,353577000
H	-4,753765000	-0,265847000	1,437184000
H	-5,884865000	-0,663436000	0,160161000
C	5,059491000	-2,442042000	-0,103574000
H	4,726665000	-3,185637000	0,628262000
H	5,851092000	-2,884876000	-0,714688000
H	4,212137000	-2,208326000	-0,754542000
C	-3,597669000	-2,334007000	0,608714000
H	-3,131421000	-3,099728000	-0,014218000
H	-4,548226000	-2,754952000	0,963271000
C	-3,411265000	2,242443000	1,495770000
H	-3,816570000	1,437303000	2,110755000
H	-4,051657000	3,119573000	1,671193000
C	-2,722345000	-2,031517000	1,821556000
H	-3,208570000	-1,279940000	2,446466000
H	-2,651488000	-2,943640000	2,432491000
C	-1,252506000	0,483577000	2,963796000
H	-2,320684000	0,447014000	3,183332000
H	-0,763595000	0,870371000	3,869426000
O	-0,739736000	0,224312000	-2,772325000
H	-0,143659000	0,963822000	-2,979587000
H	-0,268256000	-0,609469000	-2,947951000
C	-5,457464000	-2,387230000	-1,684128000
C	-6,461622000	-3,057469000	-1,739001000
H	-3,389434000	-2,121696000	-2,039372000
H	-4,365122000	-0,677076000	-2,240925000
H	-7,346451000	-3,653297000	-1,791957000

$[Y(L_2)^{\bullet}]^+$ (nine-coordinate with bound chloride) :

Y	1.472103000	-0.262521000	-0.480643000
O	-0.911684000	-0.369271000	0.077865000
O	0.898545000	1.711732000	-1.417398000
O	-7.484541000	0.766404000	-0.878543000
O	1.191976000	-2.559110000	-0.659701000
N	-6.402984000	0.301435000	-0.361721000
N	0.838094000	-1.280685000	2.017759000
N	3.481894000	-1.601193000	0.585384000
N	-2.710620000	-1.119348000	1.248676000
H	-3.020024000	-1.697453000	2.022612000

N	0.938548000	1.653255000	1.366216000
N	3.675372000	1.393467000	-0.008779000
O	2.050337000	-4.629853000	-0.850670000
O	0.173449000	3.841679000	-1.416363000
C	-0.486512000	-1.908177000	1.870239000
H	-0.948614000	-2.133895000	2.842024000
H	-0.362664000	-2.849414000	1.326930000
C	-3.744221000	-0.425457000	0.470118000
H	-3.204010000	0.232293000	-0.212548000
C	-1.401385000	-1.059686000	1.004913000
C	0.422796000	2.775409000	-0.850516000
C	3.525816000	-2.887971000	-0.157363000
H	4.062559000	-3.657563000	0.413146000
H	4.070776000	-2.722204000	-1.089849000
C	2.149731000	-3.432561000	-0.578166000
C	-5.699089000	-0.758160000	-1.161780000
C	-4.642904000	0.386482000	1.407216000
H	-5.114553000	-0.303128000	2.119769000
H	-4.044534000	1.094280000	1.991166000
C	-4.602000000	-1.428486000	-0.308298000
H	-3.972736000	-2.035125000	-0.968598000
H	-5.071580000	-2.113147000	0.409911000
C	-5.742800000	1.175011000	0.667125000
C	1.852070000	-2.305609000	2.382858000
H	1.606955000	-3.232797000	1.864326000
H	1.811454000	-2.527647000	3.459554000
C	0.089147000	2.633038000	0.645178000
H	0.118966000	3.620872000	1.123548000
H	-0.945748000	2.279851000	0.684443000
C	0.221792000	1.112692000	2.551921000
H	-0.829679000	0.988973000	2.291672000
H	0.248447000	1.831430000	3.385046000
C	-5.114287000	-0.125997000	-2.443467000
H	-5.895140000	0.435767000	-2.963952000
H	-4.747753000	-0.913825000	-3.110262000
H	-4.281988000	0.553003000	-2.236080000
C	-6.747499000	-1.814989000	-1.547899000
H	-7.228051000	-2.228895000	-0.655055000
H	-6.258754000	-2.632240000	-2.088416000
H	-7.518188000	-1.381378000	-2.188152000
C	4.299511000	1.841180000	-1.289405000
C	-6.824365000	1.592548000	1.677649000
H	-6.367305000	2.186236000	2.476164000
H	-7.295730000	0.712102000	2.126970000
H	-7.597686000	2.191431000	1.192726000
C	4.753024000	-0.845291000	0.392709000
H	5.008645000	-0.910659000	-0.666453000
H	5.571270000	-1.316236000	0.958662000
C	4.654741000	0.622409000	0.805535000
H	4.368230000	0.705422000	1.855257000
H	5.653291000	1.075131000	0.728802000
C	-5.173691000	2.434386000	-0.021370000

H	-4.845766000	3.153995000	0.736314000
H	-5.952244000	2.901303000	-0.631317000
H	-4.318412000	2.211793000	-0.665945000
C	3.197662000	2.598147000	0.720998000
H	2.752878000	3.273173000	-0.013955000
H	4.044215000	3.139826000	1.166640000
C	3.269481000	-1.865167000	2.033526000
H	3.519531000	-0.960762000	2.589605000
H	3.969205000	-2.641714000	2.378408000
C	2.195976000	2.299129000	1.826045000
H	2.656518000	1.657347000	2.579919000
H	1.961149000	3.247319000	2.334964000
C	0.808424000	-0.207343000	3.046066000
H	1.831398000	-0.046699000	3.389588000
H	0.237886000	-0.537672000	3.927386000
O	-0.105928000	-0.986782000	-2.378394000
H	0.128852000	-1.920240000	-2.193267000
H	-0.960713000	-0.859884000	-1.928819000
C	5.421999000	2.780191000	-1.131897000
C	6.350989000	3.545464000	-1.014561000
H	3.508578000	2.296834000	-1.892312000
H	4.624908000	0.957855000	-1.840654000
H	7.168901000	4.224817000	-0.915002000
Cl	2.999965000	-0.772012000	-2.770659000

$[Y(L_2)^{\bullet}]^+$ (nine-coordinate with bound triflate) :

Y	-0.967595000	-0.369857000	0.231180000
O	1.451104000	-0.470602000	0.372558000
O	-0.626234000	0.417884000	-1.905349000
O	7.675249000	1.727223000	-0.831113000
O	-0.723318000	0.147117000	2.436262000
N	6.724772000	1.031005000	-0.316305000
N	0.057917000	-2.570464000	1.519430000
N	-2.790568000	-1.546743000	1.726716000
N	3.423422000	-1.188529000	1.242470000
H	3.866137000	-1.820423000	1.901123000
N	-0.160441000	-2.289829000	-1.469703000
N	-3.079275000	-1.351364000	-1.297379000
O	-1.544918000	0.428209000	4.512325000
O	0.138654000	0.270796000	-4.016996000
C	1.345543000	-2.143088000	2.095183000
H	1.969680000	-2.998382000	2.390851000
H	1.142949000	-1.548291000	2.990506000
C	4.295685000	-0.280855000	0.486115000
H	3.642578000	0.218546000	-0.231150000
C	2.094060000	-1.211512000	1.157383000
C	-0.030818000	-0.188322000	-2.887450000
C	-2.939708000	-0.661550000	2.909894000
H	-3.370634000	-1.204622000	3.761335000
H	-3.638119000	0.139953000	2.653816000
C	-1.630787000	0.021845000	3.351528000

C	5.853496000	1.748153000	0.676922000
C	5.384172000	-1.076065000	-0.239285000
H	5.983744000	-1.610556000	0.509493000
H	4.933582000	-1.832117000	-0.891253000
C	4.954473000	0.739058000	1.420397000
H	4.191860000	1.297110000	1.974357000
H	5.557216000	0.194773000	2.159101000
C	6.313919000	-0.188645000	-1.092974000
C	-0.884517000	-2.964051000	2.602147000
H	-0.722662000	-2.305517000	3.455836000
H	-0.673438000	-3.986241000	2.949021000
C	0.561377000	-1.566213000	-2.545198000
H	0.635650000	-2.171534000	-3.458200000
H	1.579954000	-1.369839000	-2.196090000
C	0.741210000	-3.272865000	-0.810043000
H	1.735125000	-2.829915000	-0.735523000
H	0.852500000	-4.175552000	-1.429461000
C	5.018765000	2.819430000	-0.057319000
H	5.680215000	3.450167000	-0.657979000
H	4.502179000	3.451358000	0.672942000
H	4.264044000	2.384080000	-0.718932000
C	6.782670000	2.433970000	1.692961000
H	7.441387000	1.701848000	2.172105000
H	6.179790000	2.916698000	2.469035000
H	7.400166000	3.190549000	1.205120000
C	-3.889764000	-0.225379000	-1.848419000
C	7.592720000	-0.976325000	-1.424525000
H	7.325272000	-1.901609000	-1.945456000
H	8.135281000	-1.238138000	-0.509995000
H	8.253532000	-0.390026000	-2.065788000
C	-4.080410000	-1.654760000	0.987036000
H	-4.512592000	-0.653181000	0.939686000
H	-4.790543000	-2.288373000	1.539673000
C	-3.920560000	-2.215055000	-0.425528000
H	-3.472287000	-3.209700000	-0.392191000
H	-4.918620000	-2.346862000	-0.867038000
C	5.630090000	0.249461000	-2.406256000
H	5.477499000	-0.621392000	-3.052528000
H	6.269627000	0.964835000	-2.931077000
H	4.655924000	0.717414000	-2.237382000
C	-2.502535000	-2.124710000	-2.428544000
H	-2.191566000	-1.410485000	-3.193961000
H	-3.269632000	-2.761061000	-2.892234000
C	-2.342553000	-2.899012000	2.157810000
H	-2.506210000	-3.594941000	1.333942000
H	-2.972163000	-3.252287000	2.988765000
C	-1.332704000	-3.013876000	-2.029685000
H	-1.664480000	-3.747571000	-1.292603000
H	-1.023028000	-3.588652000	-2.916438000
C	0.244452000	-3.699694000	0.569315000
H	-0.712653000	-4.214616000	0.471767000
H	0.948865000	-4.436227000	0.984335000

O	0.211279000	1.888777000	0.150796000
H	1.131265000	1.583660000	0.249167000
H	0.085479000	1.977384000	-0.814843000
C	-4.954267000	-0.633946000	-2.779430000
C	-5.835059000	-0.959063000	-3.541282000
H	-3.198594000	0.463123000	-2.342006000
H	-4.328506000	0.323697000	-1.015076000
H	-6.612288000	-1.242341000	-4.216636000
O	-2.639816000	1.276945000	0.339146000
S	-3.337239000	2.529771000	0.802546000
O	-4.785802000	2.317260000	0.976582000
O	-2.639420000	3.265137000	1.868275000
C	-3.189836000	3.614717000	-0.700995000
F	-3.765605000	4.798859000	-0.461835000
F	-1.901523000	3.817659000	-1.010320000
F	-3.796334000	3.045144000	-1.752282000
